# Identification of m^7^G regulator-mediated RNA methylation modification patterns and related immune microenvironment regulation characteristics in heart failure

**DOI:** 10.1186/s13148-023-01439-3

**Published:** 2023-02-13

**Authors:** Chaoqun Ma, Dingyuan Tu, Qiang Xu, Yan Wu, Xiaowei Song, Zhifu Guo, Xianxian Zhao

**Affiliations:** 1 Cardiovascular Research Institute and Department of Cardiology, General Hospital of Northern Theater Command, Shenyang, 110000 Liaoning China; 2 Department of Cardiology, Changhai Hospital, Naval Medical University, 168 Changhai Rd, Shanghai, 200433 China; 3 Department of Cardiology, Navy 905 Hospital, Naval Medical University, Shanghai, 200052 China

**Keywords:** Heart failure, N^7^-methylguanosine, Machine learning, Unsupervised clustering, Immune infiltration, Bioinformatic analysis

## Abstract

**Background:**

N^7^-methylguanosine (m^7^G) modification has been reported to regulate RNA expression in multiple pathophysiological processes. However, little is known about its role and association with immune microenvironment in heart failure (HF).

**Results:**

One hundred twenty-four HF patients and 135 nonfailing donors (NFDs) from six microarray datasets in the gene expression omnibus (GEO) database were included to evaluate the expression profiles of m^7^G regulators. Results revealed that 14 m^7^G regulators were differentially expressed in heart tissues from HF patients and NFDs. Furthermore, a five-gene m^7^G regulator diagnostic signature, NUDT16, NUDT4, CYFIP1, LARP1, and DCP2, which can easily distinguish HF patients and NFDs, was established by cross-combination of three machine learning methods, including best subset regression, regularization techniques, and random forest algorithm. The diagnostic value of five-gene m^7^G regulator signature was further validated in human samples through quantitative reverse-transcription polymerase chain reaction (qRT-PCR). In addition, consensus clustering algorithms were used to categorize HF patients into distinct molecular subtypes. We identified two distinct m^7^G subtypes of HF with unique m^7^G modification pattern, functional enrichment, and immune characteristics. Additionally, two gene subgroups based on m^7^G subtype-related genes were further discovered. Single-sample gene-set enrichment analysis (ssGSEA) was utilized to assess the alterations of immune microenvironment. Finally, utilizing protein–protein interaction network and weighted gene co-expression network analysis (WGCNA), we identified UQCRC1, NDUFB6, and NDUFA13 as m^7^G methylation-associated hub genes with significant clinical relevance to cardiac functions.

**Conclusions:**

Our study discovered for the first time that m^7^G RNA modification and immune microenvironment are closely correlated in HF development. A five-gene m^7^G regulator diagnostic signature for HF (NUDT16, NUDT4, CYFIP1, LARP1, and DCP2) and three m^7^G methylation-associated hub genes (UQCRC1, NDUFB6, and NDUFA13) were identified, providing new insights into the underlying mechanisms and effective treatments of HF.

**Supplementary Information:**

The online version contains supplementary material available at 10.1186/s13148-023-01439-3.

## Background

Heart failure (HF), a complex clinical syndrome, is the terminal stage of various cardiovascular diseases including myocardial infarction, hypertension, myocarditis, cardiomyopathy, and arrhythmias [1]. Despite the current advances in medical treatment and interventional therapy, the prognosis of HF patients remains poor, which highlights the urgency for further exploration of the molecular and cellular mechanisms underlying HF. Emerging evidence indicates that inflammatory activation and immune infiltration are associated tightly with HF onset, progression and prognosis [2, 3]. To investigate the immune microenvironment alterations and key regulators in the development of HF may provide new directions for its accurate diagnosis, early intervention and precision therapy.

RNA modifications, types of post-transcriptional regulation, have emerged as key regulators for RNA structural stability and cell metabolism [4, 5]. N^7^ methylguanosine (m^7^G), one of the positively charged base modifications, has been recently reported to be an essential modification at the 5’ cap of eukaryotic mRNA, regulating mRNA export, translation, and splicing [6]. Besides, m^7^G also occurs in position 46 of transfer RNA (tRNA) variable loop [7] and eukaryotic 18S ribosomal RNA (rRNA) [8]. Previous studies have shown that aberrant m^7^G RNA modification is associated with the progression of various pathological processes, such as lung cancers [9], hepatocarcinoma [10], and ischemic disorders [11]. Chen et al. reported that METTL1 could promote hepatocarcinogenesis via m^7^G tRNA modification-dependent translation control [10]. Zhao et al. emphasized a critical link between mRNA m^7^G alteration and post-ischemic injury in peripheral arterial diseases [11]. However, the role of m^7^G in HF development, especially the immune responses against cardiac inflammation, has been poorly studied.

With the rapid development of microarray and high-throughput sequencing technology [12, 13], an increasing volume of RNA-sequencing (RNA-seq) and microarray datasets of HF have been uploaded in the gene expression omnibus (GEO) database, providing opportunities for bioinformatics data mining of marker genes and epigenetic changes associated with HF [14]. However, one of the key challenges of data processing is dealing with the feature dimensionality and redundancy of the data. To resolve this issue, machine learning algorithms are increasingly used to ascertain classifiers for feature selection and establish robust diagnostic or prognostic prediction models of different diseases [15, 16]. For example, Deng et al. used least absolute shrinkage and selection operator (LASSO) regression and support vector machine-recursive feature elimination (SVM-RFE) to perform feature selection to screen diagnostic markers for osteoarthritis [17]. Based on this, the cross-combination of machine learning may help in bioinformatics data mining and analysis of HF-related m^7^G modifications.

In this study, we systematically evaluate the modification pattern of m^7^G regulators in HF based on six publicly available HF microarray datasets (GSE16499 [18], GSE26887 [19], GSE42955 [20], GSE57338 [21], GSE76701 [22], and GSE79962 [23]). In addition, through the cross-combination of three feature selection algorithms, a five-gene m^7^G regulator diagnostic signature was established that can well distinguish HF samples and nonfailing donors (NFDs). Moreover, we clustered HF samples based on the expression profiles of m^7^G regulators and discovered two distinct m^7^G modification subtypes of HF with different immune characteristics and biological functions. Besides, three m^7^G methylation hub genes were finally identified through clinical traits analyses. These findings above indicate that m^7^G modification patterns have significant impacts on the immune microenvironment of HF development.

## Results

### Landscape of m^7^G methylation regulators in HF

**Fig. 1:**
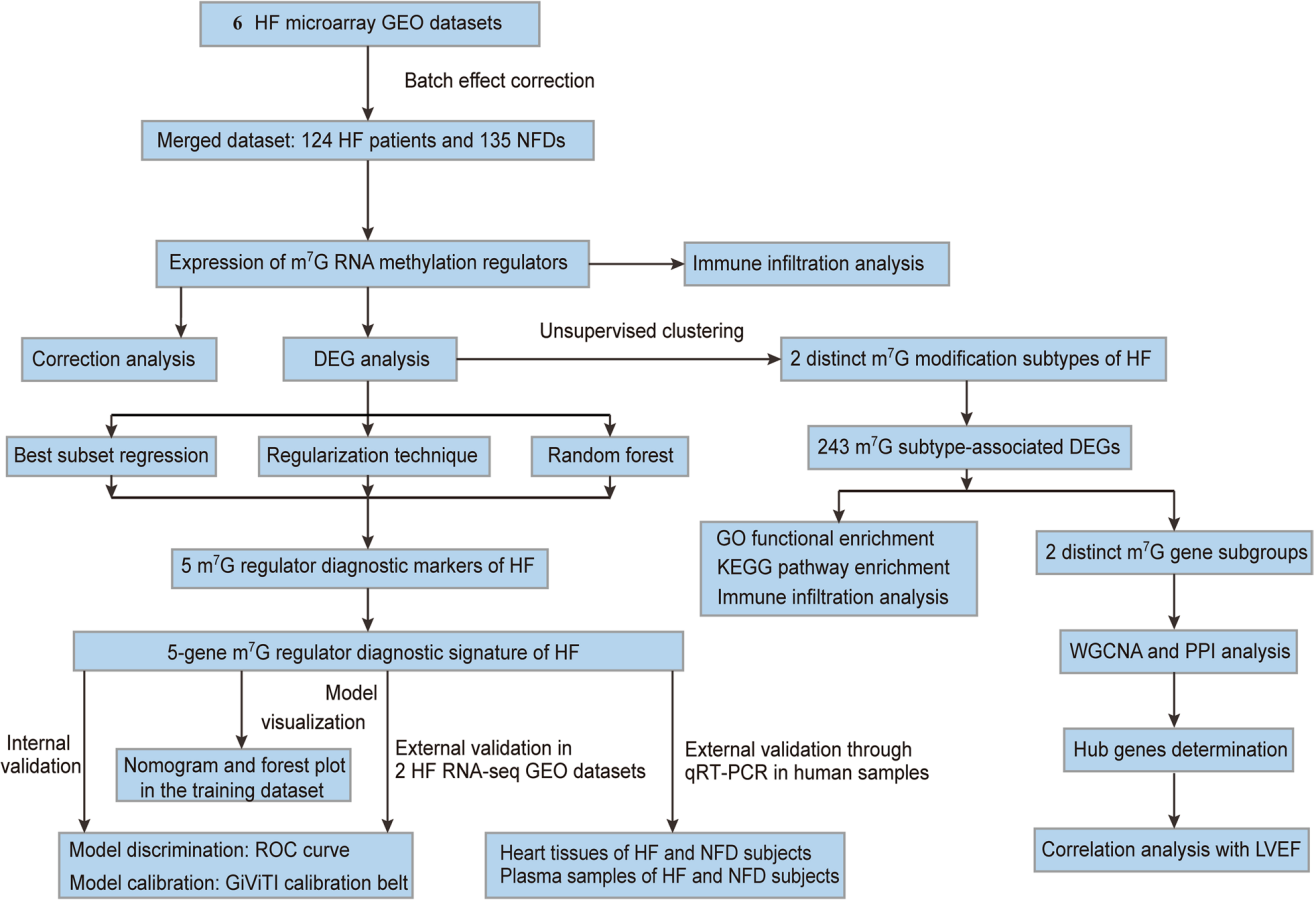
Study flow diagram. HF, heart failure; *NFD* Nonfailing donor, *GEO* Gene expression omnibus, *NFDs* Nonfailing donors, m^7^G, N^7^-methylguanosine, *DEG* Differentially expressed gene, *qRT-PCR* Quantitative reverse-transcription polymerase chain reaction, *GO* Gene ontology, *KEGG* Kyoto Encyclopedia of Genes and Genomes, *RNA-seq* RNA-sequencing, *WGCNA* Weighted gene co-expression network analysis, *PPI* Protein–protein interaction, *LVEF* Left ventricular ejection fraction, *ROC* Receiver operating characteristic

**Fig. 2:**
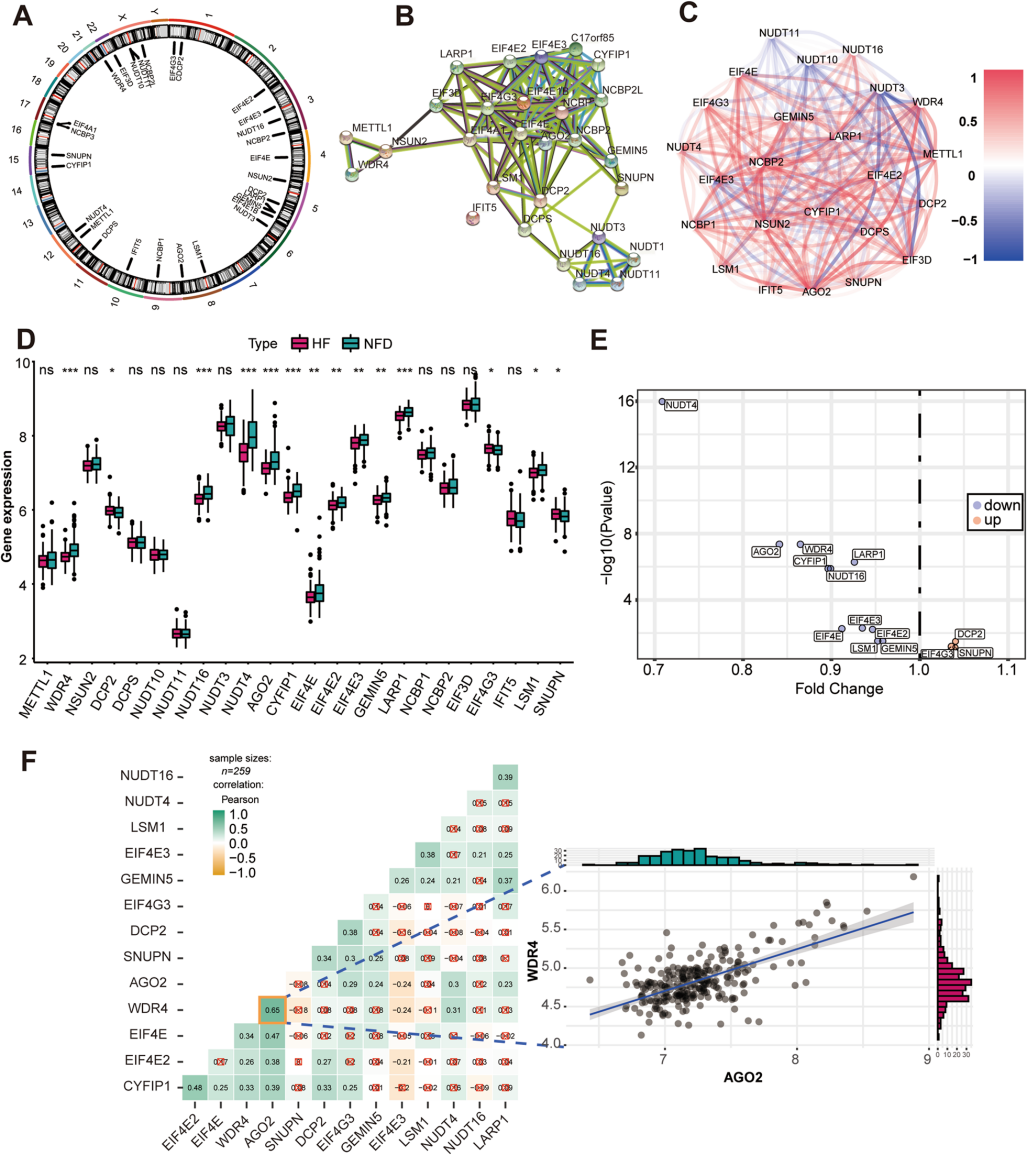
Landscape of m^7^G RNA methylation regulators in HF. **A** Circus plot of chromosome distributions of the 29 m^7^G regulators. **B** protein–protein interaction (PPI) network among the 29 m^7^G RNA methylation regulators. **C** Correlations among the 24 m^7^G regulators in heart samples from HF patients and NFDs. A positive correlation is indicated by red, while a negative correlation is indicated by blue. **D** Expression profiles of m^7^G RNA methylation regulators between HF patients and NFDs. ns = not significant, *p < 0.05, **p < 0.01, and ***p < 0.001 vs. the NFD group. **E** Volcano plot showing the differential expression of 14 m^7^G regulators between HF patients and NFDs. **F** Correlation analysis among 14 differentially expressed m^7^G regulators in HF patients. ☒ in red stands for nonsignificant at *p* < 0.05. The scatter plot demonstrated the m^7^G regulators pair with the highest differential correlation, WDR4 and AGO2 with the most positive correlation

The overall research strategy is presented in Fig. 1. In this study, 29 m^7^G RNA methylation regulators were harvested from the MSigDB team, and Fig. 2A displays the location of these genes on chromosomes. To examine the interactivity of the m^7^G regulators, PPI network was created through the STRING website. Results showed that the 29 regulators had a strong connection, indicating that they may function as a complex (Fig. 2B**)**. Expression analysis then revealed that 24 of the m^7^G RNA methylation regulators were identified in human heart samples (Fig. 2C**)**. Among them, 14 regulators were observed with significant differentially expression in heart tissues from HF patients and NFDs (*p* < 0.05, Fig. 2D–E**)**. NUDT4 had the largest fold change and the most statistically significant change. In the correlation analysis, we found that there were close correlations among the 14 differentially expressed m^7^G regulators, which laid the foundation for the subsequent m^7^G cluster analysis, the WDR4 and AGO2 were the most correlated regulators in expression, suggesting that they function together (Fig. 2F).

### Determination of m^7^G regulator diagnostic markers for HF

**Fig. 3:**
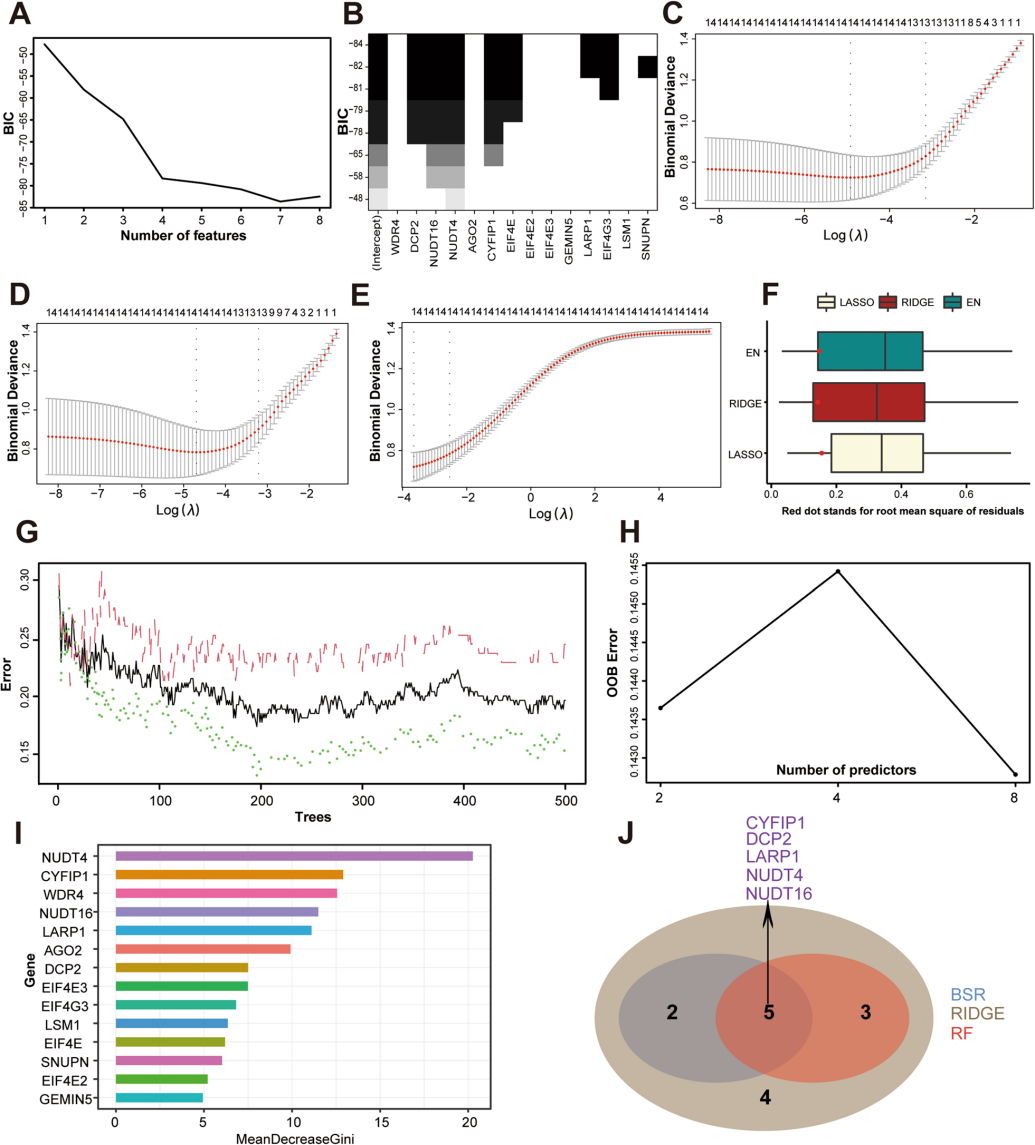
Screening m^7^G regulator diagnostic markers by three feature selection algorithms.** A** Bayesian information criterion score by feature inclusion of best subset regression (BSR) analysis. **B** Model performance based on different feature subsets in BSR analysis. **C** Least absolute shrinkage and selection operator (LASSO) regression algorithm to identify diagnostic markers. **D** RIDGE regression algorithm to identify diagnostic markers. **E** Elastic net (EN) regression algorithm to identify diagnostic markers for HF. **F** Root mean squared error (RMSE) of three regularization technique models in the internal validation dataset. **G** Out-of-bag (OOB) error rate of the random forest (RF) model. **H** Search for the optimal value of mtry for RF model. **I** Variable importance plot for the RF model. The features are ranked by the mean decrease in classification accuracy when they are permuted. The more the Gini coefficient decreases on average, the more important the variable is.** J** Venn diagram showing the intersected genes of BSR analysis, RIDGE regression and RF algorithm

To develop a m^7^G regulator diagnostic signature to predict HF, three machine learning techniques with feature selection were applied. First, best subset regression (BSR) analysis identified the subset of seven features (CYFIP1, DCP2, EIF4E, EIF4G3, LARP1, NUDT16, and NUDT4) with the lowest Bayesian information criterion (BIC) score (BIC = − 83.60938, Fig. 3A–B). Second, for regularization technique, we obtained and compared the coefficient profile plots of LASSO, ridge (RIDGE), and elastic net (EN) regression (Fig. 3C–E). As shown in Fig. 3F, the optimal regularization technique model is the RIDGE regression model with the minimum value of root mean squared error (RMSE) in the internal validation dataset. Third, we established a RF prediction model with all m^7^G regulators, which achieved an accuracy rate of 85.8% with 196 trees and 8 mtry (Fig. 3G–H). Feature ranking of the random forest (RF) algorithm was generated according to the mean decrease of Gini index. Figure 3I showed that NUDT4, CYFIP1, WDR4, NUDT16, LARP1, AGO2, DCP2, and EIF4E3 were the most important features for HF risk prediction. Taken together, intersection of results from BSR analysis, RIDGE regression, and RF algorithm revealed that NUDT16, NUDT4, CYFIP1, LARP1, and DCP2 were potential m^7^G regulator diagnostic markers for HF (Fig. 3J).

### Development and validation of the m^7^G regulator diagnostic signature for HF

**Fig. 4:**
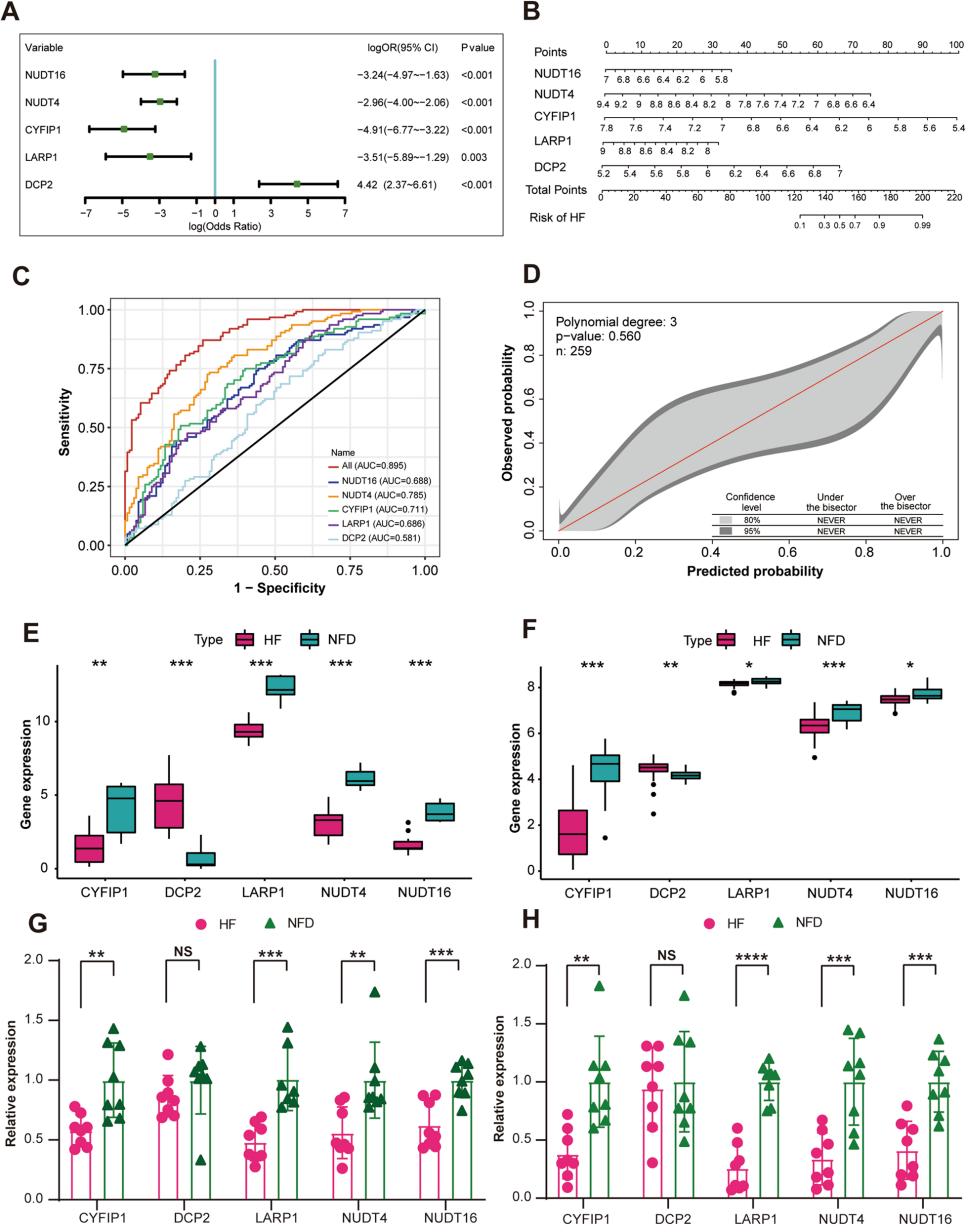
Development and validation of the m^7^G regulator diagnostic signature for HF.** A** Forest plot of the multivariate logistic regression analysis to investigated the relationship between the five m^7^G regulator diagnostic markers and HF. **B** Nomogram of the five-gene m^7^G regulator diagnostic signature for HF probability. **C** receiver operating characteristic (ROC) curve of the five-gene m^7^G regulator diagnostic signature in the merged dataset. **D** The GiViTi calibration belts of the five-gene m^7^G regulator diagnostic signature in the merged dataset. **E** The expression profiles of the five m^7^G regulators diagnostic markers in the external validation dataset GSE116250. **p < 0.01, and ***p < 0.001 vs. the NFD group. **F** The expression profiles of five m^7^G regulators diagnostic markers in the external validation dataset GSE46224. *p < 0.05, **p < 0.01, and ***p < 0.001 vs. the NFD group. **G** Validation of the 5 m^7^G regulators diagnostic markers expression (CYFIP1, DCP2, LARP1, NUDT4, and NUDT16) by quantitative real-time reverse-transcription PCR (qRT-PCR) using human heart tissues from HF patients and NFDs. Data are presented with mean ± standard deviation (SD), n = 8. **p < 0.01, and ***p < 0.001 vs. the NFD group. NS, no significance. **H** Validation of the 5 m^7^G regulators diagnostic markers expression by qRT-PCR using plasma samples from HF patients and NFDs. Data are presented with mean ± SD, n = 8. NS**p < 0.01, ***p < 0.001, and ****p < 0.0001 vs. the NFD group. NS, no significance

To estimate the association between the expression of the five m^7^G regulator diagnostic markers and HF, we constructed a logistic regression model. Multivariate logistic regression analysis demonstrated that all of the five markers expression were independently associated with HF, as visualized by the forest plot (Fig. 4A, Additional file 1: Table S1) and nomogram (Fig. 4B). The established five-gene m^7^G regulator diagnostic signature model exhibited an AUC of 0.895 (95% CI, 0.859–0.931) in the merged dataset, indicating that it performed well in classifying HF and NFD samples (Fig. 4C). In the independent external validation dataset (GSE46224 and GSE116250), the five-gene diagnostic signature model yielded the AUC of 0.908 (95% CI 0.786–0.999) and 0.950 (95% CI 0.889–0.999), respectively (Additional file 1: Fig. S1A–B). Additionally, the 95% CI region of GiViTI calibration belt did not cross the 45-degree diagonal bisector line in the merged dataset and two external validation datasets (*p* = 0.560 for the merged dataset, *p* = 0.810 for GSE46224 [24], and *p* = 0.523 for GSE116250 [25]), indicating a good fit between the predicted and observed probabilities for HF (Fig. 4D, Additional file 1: Fig. S1C–D). Meanwhile, differential expressions of the m^7^G regulator diagnostic markers were also verified in two external validation datasets, which further demonstrated their diagnostic capacity for HF (Fig. 4E–F). In addition to the microarray datasets, we conducted quantitative reverse-transcription polymerase chain reaction (qRT-PCR) experiments to further validate the expression of the m^7^G regulator diagnostic markers using heart tissues and plasma samples from HF patients or NFDs. As described in Fig. 4G–H, four of the m^7^G regulator diagnostic markers (CYFIP1, LARP1, NUDT4, and NUDT16) were significantly downregulated in the heart tissues or plasma samples of HF patients compared with NFDs (*p* < 0.05), which was consistent with the bioinformatics analysis results, while the differential expression of DCP2 between HF and NFDs was not statistically significant. Overall, the established five-gene m^7^G regulator diagnostic signature showed excellent diagnostic performance for HF.

### m^7^G regulators are associated with immune characteristics of HF

**Fig. 5:**
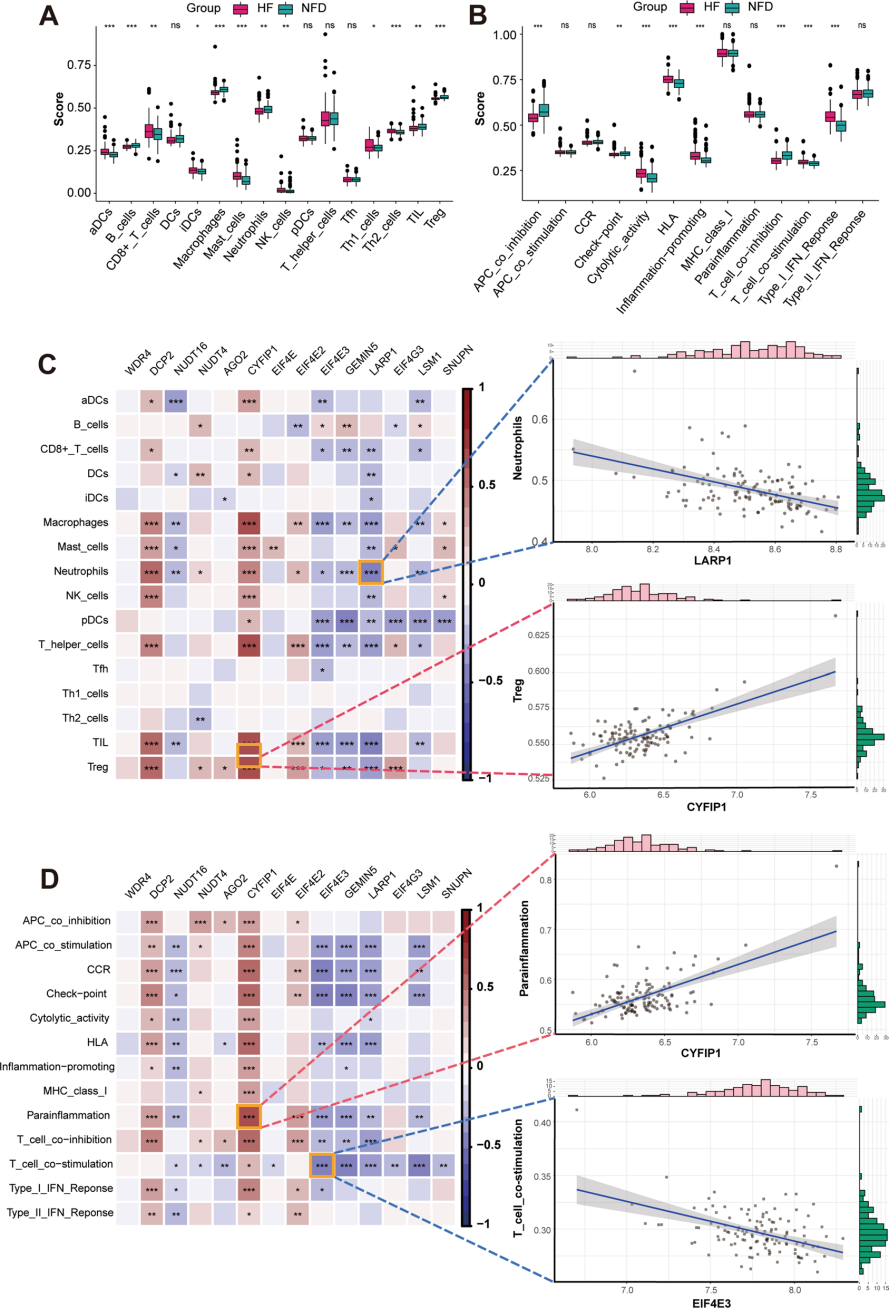
m^7^G regulators are associated with immune characteristics of HF. **A** The infiltrating scores of 16 immune cells in cardiac tissues from HF patients and NFDs. ns = not significant, *p < 0.05, **p < 0.01, and ***p < 0.001 vs. the NFD group. **B** The infiltrating scores of 13 immune-related functions in cardiac tissues from HF patients and NFDs. ns = not significant, **p < 0.01, and ***p < 0.001 vs. the NFD group. **C** Correlations between 14 differentially expressed m^7^G regulators and 16 immune cells infiltrations in HF, as visualized by heat map. The two scatter plots displayed the most positively or negatively correlated immune cells-m^7^G regulator pair. **D** Correlations between 14 differentially expressed m^7^G regulators and 13 immune-related functions in HF, as visualized by heat map. The two scatter plots displayed the most positively or negatively correlated immune function-m^7^G regulator pair

To further elucidate the association between m^7^G regulators and immune characteristics, we performed correlation analysis between them. The infiltrating scores of 16 immune cells and 13 immune-related functions were quantified using the single-sample gene-set enrichment analysis (ssGSEA) algorithm. As demonstrated in Fig. 5A, the abundance of 12 immune infiltrating cells differed significantly between HF and NFDs samples, including aDCs, B cells, CD8 + T cells, iDCs, macrophages, mast cells, neutrophils, NK cells, Th1 cells, Th2 cells, TIL, and Treg. Furthermore, correlation analysis revealed that the differentially expressed m^7^G regulators are closely related to a variety of immune cell infiltrations (Fig. 5C). Of these, Treg-CYFIP1 is the most positively correlated pair, and the most negatively correlated immune cells-m^7^G regulator pair is neutrophils-LARP1, with a correlation coefficient of 0.63 and -0.47, respectively. In addition, Fig. 5B shows the eight significant expression changes of immune-related functions in HF, including APC co-inhibition, check-point, cytolytic activity, HLA, inflammation promoting, T cell co-inhibition, T cell co-stimulation, and type I IFN response. Likewise, significant correlations were observed between m^7^G regulator expression and immune-related functions (Fig. 5D). Parainflammation was most positively correlated with CYFIP1, with a correlation coefficient of 0.61. T cell co-inhibition was most negatively correlated with EIF4E3, with a correlation coefficient of -0.47. Taken together, the results indicated that immune dysregulation exists in HF and is affected by altered m^7^G RNA methylation regulators.

### Unsupervised cluster analysis of m^7^G modification patterns in HF

**Fig. 6:**
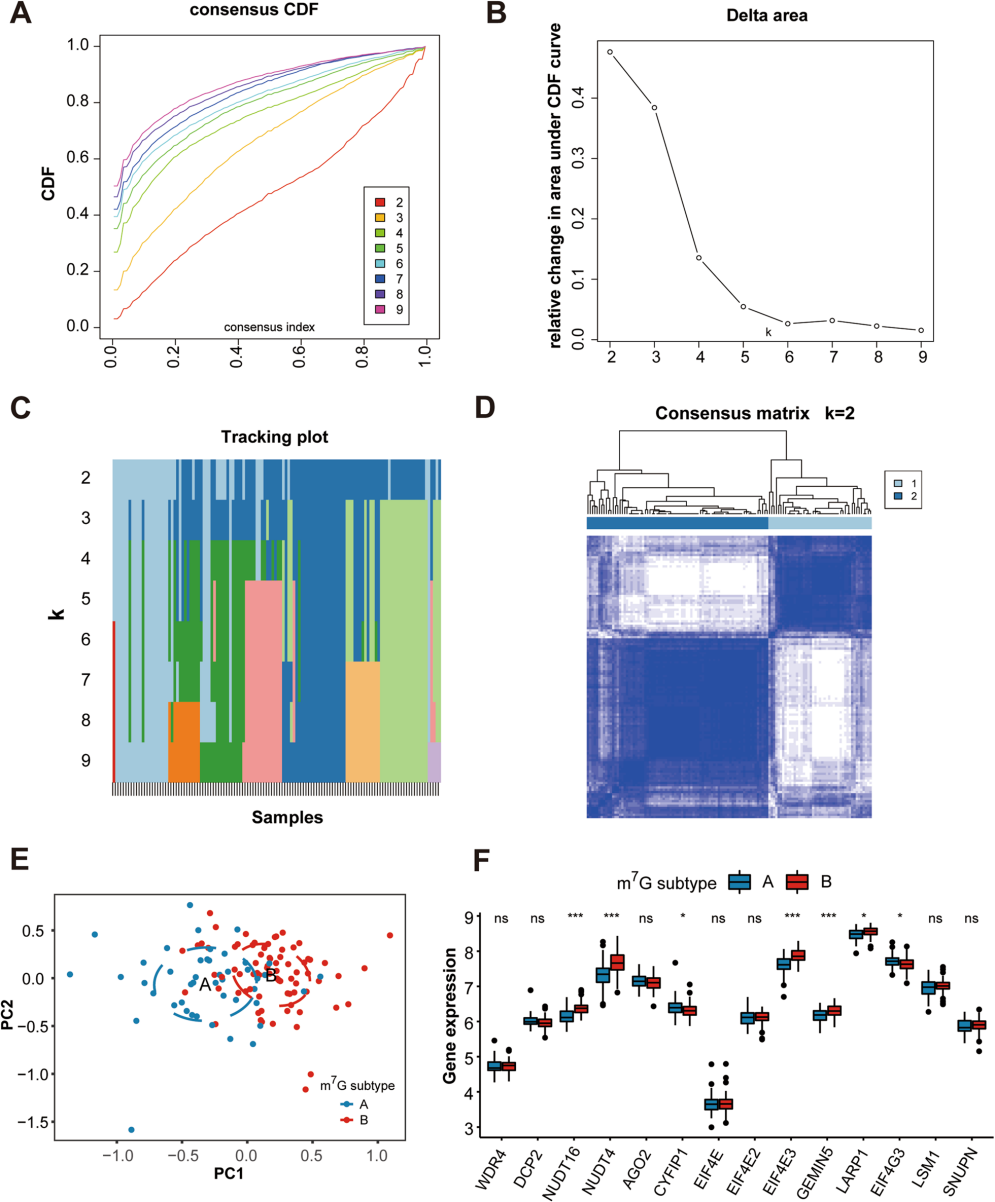
Identification of two distinct m^7^G modification subtypes across HF samples.** A** Consensus clustering model with cumulative distribution function (CDF) for *k* = 2–9. k means cluster count. **B** Relative change in the area under the CDF curve for *k* = 2–9. **C** The consensus cluster of items (in column) at *k* = 2–9 (in row). **D** Consensus matrix heatmap defining two subtypes (*k* = 2) and their correlation area. **E** Principal component analysis (PCA) showing a remarkable difference in transcriptomes between the two subtypes of HF. **F** The two m^7^G subtypes exhibit distinct expression profiles of the 14 m^7^G RNA methylation regulators

To investigate m^7^G modification patterns in HF, we conducted unsupervised consensus clustering analysis for HF samples based on the expression of 14 differentially expressed m^7^G regulators. The clustering results showed that *k* = 2 seemed to be an adequate selection, indicating that HF patients were accurately dispersed into two subtypes, subtype A (*n* = 45) and subtype B (*n* = 79) (Fig. 6A–D). Heatmaps of the matrix of co-occurrence proportions for* k* = 3 to 9 were shown in Additional file 1: Fig. S2A–G. Principal component analysis (PCA) revealed prominent differences in the expression portraits between the two subtypes (Fig. 6E). In addition, expression of 14 m^7^G regulators displayed marked heterogeneity between two subtypes (Fig. 6F). CYFIP1 and EIF4G3 were highly expressed in m^7^G subtype A, whereas NUDT16, NUDT4, EIF4E3, GEMIN5, and LARP1 were highly expressed in m^7^G subtype B. In addition, the m^7^G modification expression pattern in each HF subtype was also compared with that of NFDs. Results showed that all of the five m^7^G regulator diagnostic markers (CYFIP1, LARP1, NUDT4, NUDT16, and DCP2) were differentially expressed among the three groups (Additional file 1: Fig. S3), which further indicated that the five-gene m^7^G regulator diagnostic signature was effective to discriminate HF subgroups compared to NFDs.

### Immune signature and pathways of two distinct m^7^G subtypes

**Fig. 7:**
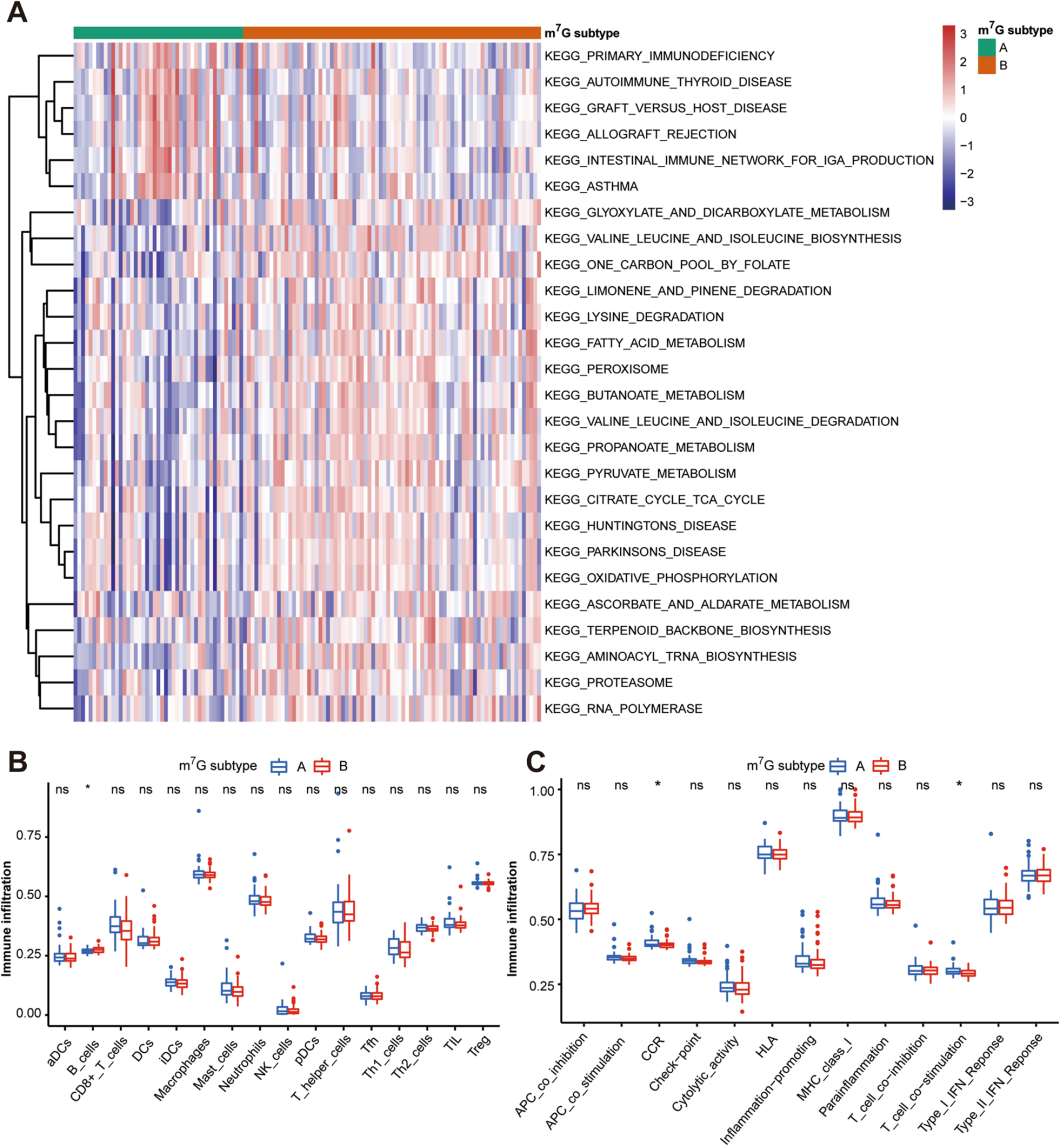
Immune signature and pathways of two distinct m^7^G subtypes. **A** Gene-set variation analysis (GSVA) of biological pathways enrichment between two m^7^G subtypes. **B** The infiltration scores of 16 immune cells between two m^7^G subtypes. ns = not significant, *p < 0.05 vs. the m^7^G subtype A. **C** The infiltration scores of 13 immune-related functions between two m^7^G subtypes. ns = not significant, *p < 0.05 vs. the m^7^G subtype A

Gene-set variation analysis (GSVA) enrichment analysis showed that compared with the B subtype, m^7^G subtype A was significantly enriched in immune fully-activated pathways, including primary immunodeficiency, autoimmune thyroid disease, graft versus host disease, allograft rejection, intestinal immune network for IgA production, and asthma (Fig. 7A). To further investigate the correlation between m^7^G subtypes and immune characteristics in HF, we compared their difference in the abundance of infiltrating immune cells and activity of immune-related functions using the ssGSEA algorithm. As shown in Fig. 7B, more infiltrating activated B cells were observed in m^7^G subtype B. For immune-related functions, m^7^G subtype A of HF exhibited significantly greater activation of CCR and T cell co-stimulation (Fig. 7C). Besides, the distribution of infiltrating immune cells and activity of immune-related functions in each HF subtype was also compared with that of NFDs (Additional file 1: Fig. S4A–B). Overall, the results indicate that two distinct m^7^G modification patterns are associated with different immune signatures and pathways, suggesting that m^7^G RNA methylation regulators may play an important role in the regulation of the HF immune microenvironment.

### Determination of HF gene subgroups based on m^7^G subtype-associated differentially expressed genes (DEGs)

**Fig. 8:**
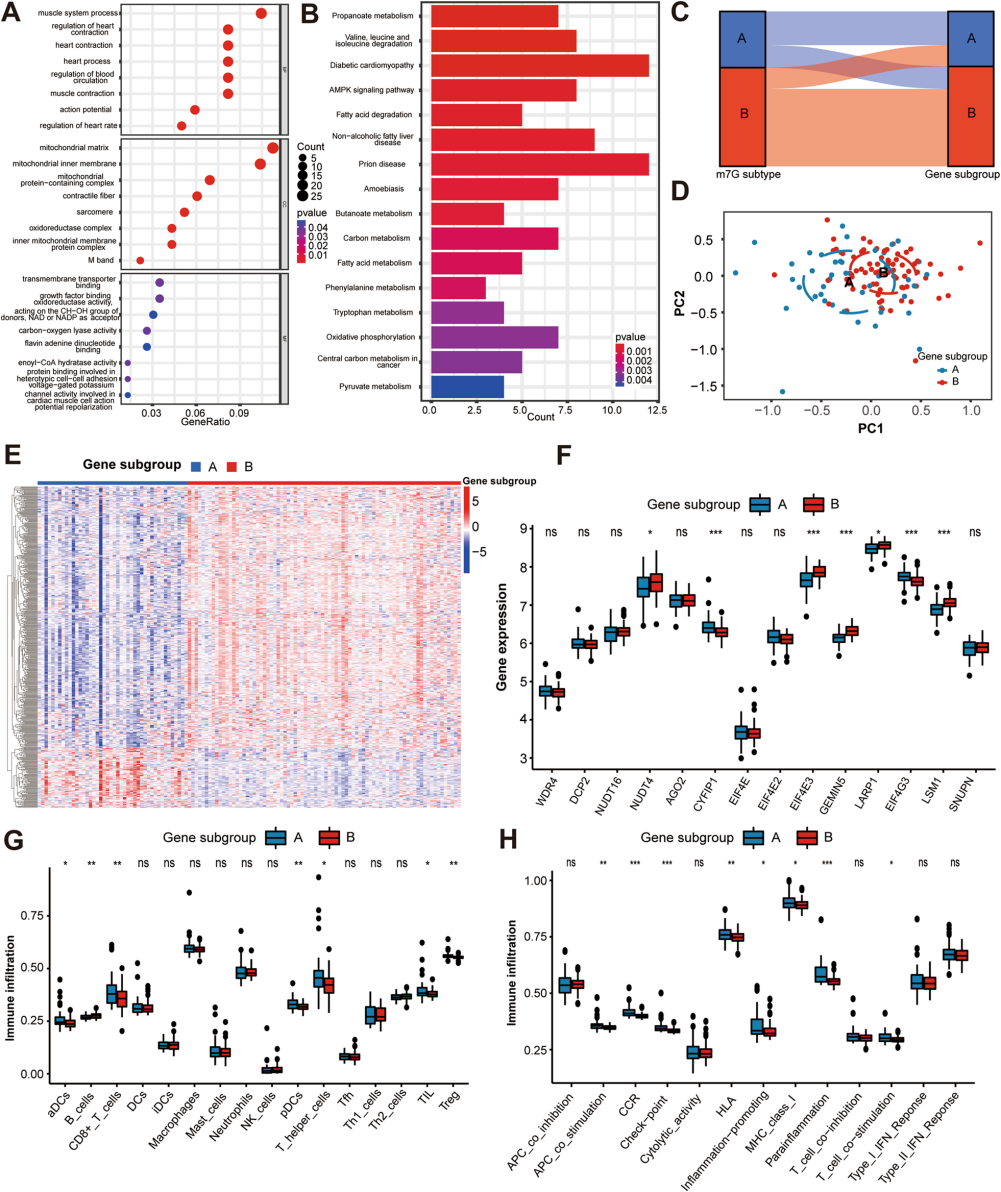
Determination of HF gene subgroups based on m^7^G subtype-associated DEGs.** A** GO enrichment analysis of DEGs between two m^7^G subtypes. BP, biological process, CC, cellular components, MF, molecular functions. **B** KEGG enrichment analysis of DEGs between two m^7^G subtypes. **C** Alluvial diagram showing the changes of m^7^G subtypes and m^7^G gene subgroups of HF. **D** PCA plot showing a remarkable difference in transcriptomes between two HF gene subgroups. **E** Heatmap of DEGs between two m^7^G gene subgroups. **F** The two HF gene subgroups exhibit distinct expression profiles of the 14 m^7^G RNA methylation regulators. ns = not significant, *p < 0.05, ***p < 0.010 vs. gene subgroup A. **G** The infiltration scores of 16 immune cells between two m^7^G gene subgroups. ns = not significant, *p < 0.05, **p < 0.01 vs. gene subgroup A.** H** The infiltration scores of 13 immune-related functions between two m^7^G gene subgroups. ns = not significant, *p < 0.05, **p < 0.01, and ***p < 0.001 vs. gene subgroup A. *DEGs* Differentially expressed genes, *GO* Gene ontology, *KEGG* Kyoto Encyclopedia of Genes and Genomes, *PCA* Principal component analysis

To explore the underlying biological functions of each m^7^G subgroup, we obtained 243 m^7^G subtype-associated DEGs in HF using the R package “limma” and performed functional enrichment analysis. Gene ontology (GO) functional and Kyoto Encyclopedia of Genes and Genomes (KEGG) pathways analysis showed that m^7^G subtype-associated DEGs were significantly enriched in heart function-associated biological processes, as well as heart-related and metabolism-related pathways, suggesting that m^7^G methylation may serves as a key factor in regulating cardiac substance and energy metabolism (Fig. 8A–B).

In addition, we further conducted clustering analysis based on the m^7^G subtype-related DEGs and classified the HF patients into two gene subgroups (subgroup A and B, Additional file 1: Fig. S5**)**. For HF patients, the alluvial diagram showed similar grouping tendency for m^7^G subtypes and gene subgroups (Fig. 8C). The heatmap and PCA revealed significant heterogeneity between samples of the two gene subgroups (Fig. 8D–E), and the expressions of m^7^G regulators showed substantial differences in two gene subgroups (Fig. 8F). Regarding the immune characteristics, the enrichment levels of aDCs, CD8 + T cells, pDCs, T helper cells, TIL, Treg were markedly higher in the gene subgroup A, while more B cells infiltrations were observed in m^7^G subtype B (Fig. 8G). Moreover, gene subgroup A of HF exhibited greater activation of eight immune-related functions, including APC co-stimulation, CCR, check-point, HLA, inflammation promoting, MHC class I, parainflammation, and T cell co-stimulation (Fig. 8H). The above results suggested that HF patients in the gene subgroup A had higher levels of immune infiltration compared with subgroup B.

### Identification of m^7^G-related hub genes and clinical correlation with cardiac function

**Fig. 9:**
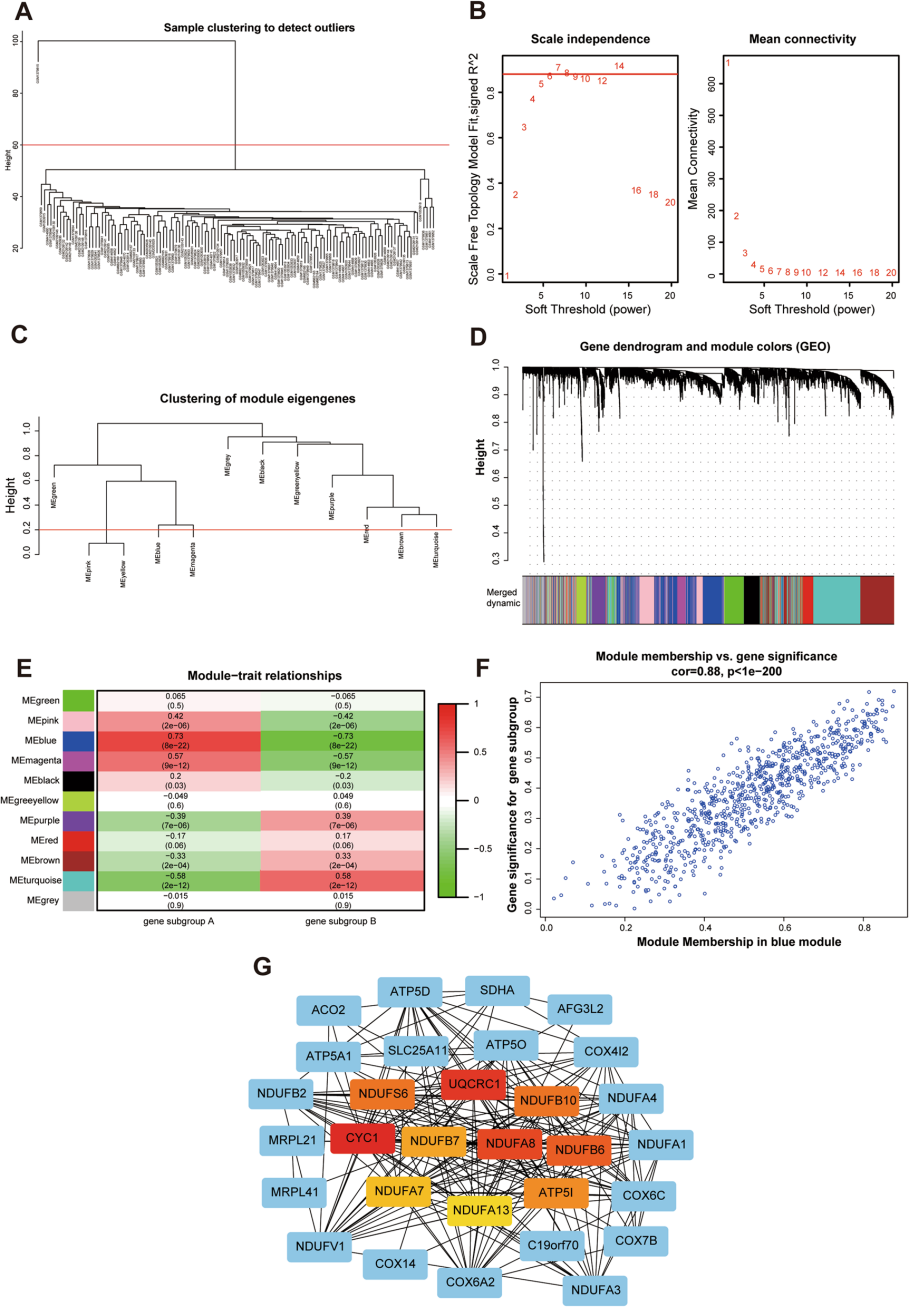
Identification of m^7^G methylation-related hub genes in HF. **A** Sample clustering was conducted based on the expression data of all HF samples. The top 25% variation genes were used for WGCNA, and outlier samples were excluded. The red line indicates the cutoff threshold (60). **B** Scale-free topology index analysis and mean connectivity of soft threshold power from 1 to 20. The red line indicates the scale-free *R*^*2*^ (0.879). **C** Clustering dendrogram of module eigengenes. The red line indicates the cut height (0.20).** D** Gene dendrogram obtained by average linkage hierarchical clustering. The genes were clustered into different modules through hierarchical clustering and merged when the correlation of the modules is > 0.8. **E** Heatmap of the correlation between module eigengenes and HF gene subgroups. **F** Correlation between module membership (X-axis) and gene significance (Y-axis) of genes from the blue module. **G** Protein–protein interaction (PPI) network of genes from the blue module. The central nodes in PPI network are marked in red, yellow, and orange

**Fig. 10:**
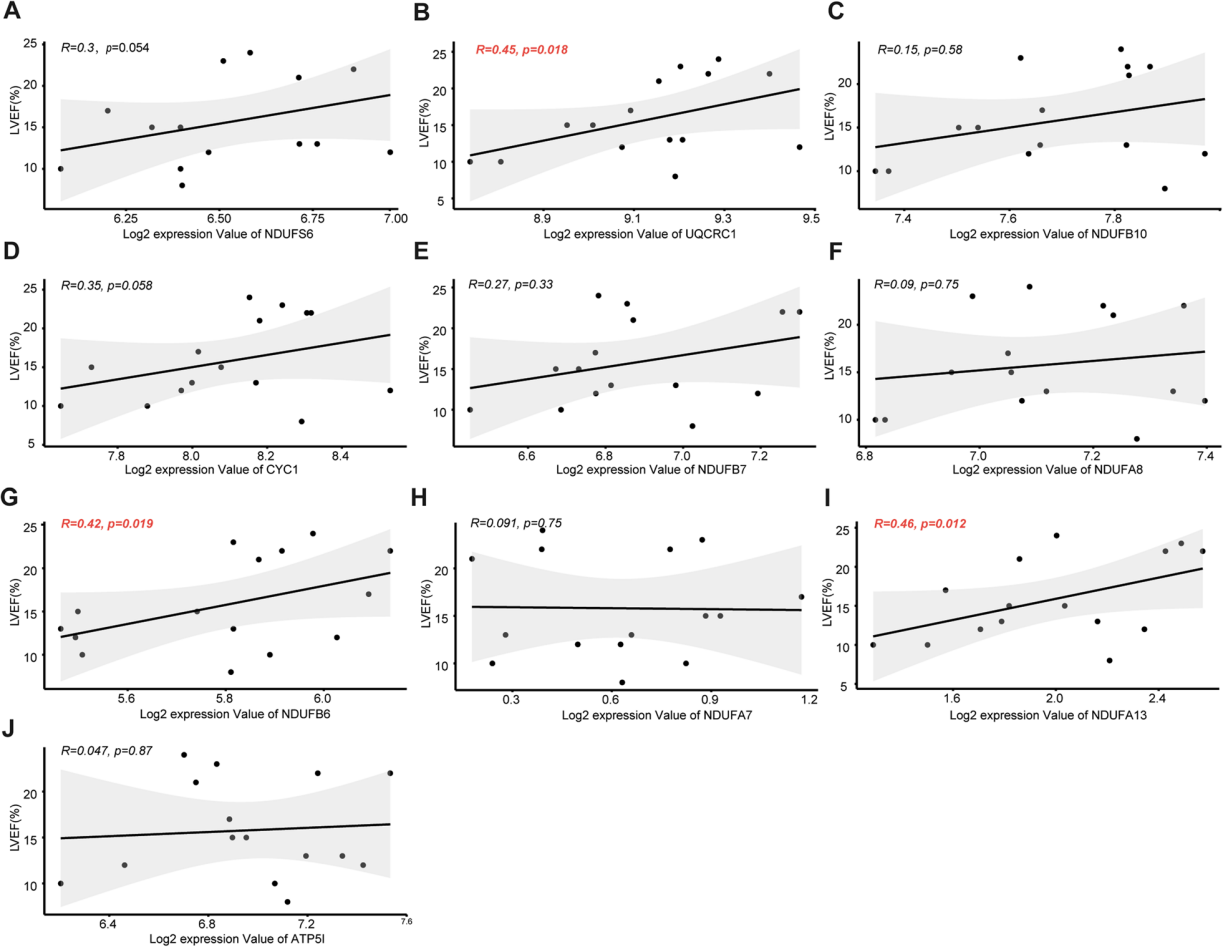
Relationship between m^7^G markers expression levels and LVEF in HF patients. *LVEF* Left ventricular ejection fraction

Figure 9A illustrated the dendrogram and traits of 124 HF samples based on weighted gene co-expression network analysis (WGCNA), and when *β* = 6 (a soft threshold), the scale-free *R*^*2*^ was 0.879 to obtain a higher average connectivity degree (Fig. 9B). Similar modules with a height cutoff value of 0.2 were merged (Fig. 9C), and 11 modules were identified by hierarchical clustering and the dynamic branch cutting (Fig. 9D). From the heatmap of module-trait correlations, the MEblue module genes were most positively correlated with m^7^G gene subgroup A of HF (*R*^*2*^ = 0.73), indicating that MEblue is a key module (Fig. 9E–F). Subsequently, 158 genes in the MEblue module were used to construct the PPI network, and hub genes were selected by cytoHubba in Cytoscape software. Finally, ten m^7^G RNA methylation modification markers (NUDFS6, UCRC1, NDUFB10, CYC1, NDUFB7, NDUFA8, NDUFB6, NDUFA7, NDUFA13, and ATP5IF1) were identified (Fig. 9G). To further illuminate the roles of these ten m^7^G markers in HF, Pearson correlation was applied to analysis the correlation between the mRNA expression of these markers and left ventricular ejection fraction (LVEF) in 15 HF patients of GSE46224. As showcased in Fig. 10, the expression of UQCRC1, NDUFB6, or NDUFA13 was positively correlated with LVEF in HF patients, indicating that these m^7^G-related genes may be involved in improving cardiac function of HF patients.

## Discussion

In recent years, the role of inflammation and immune activation in the development of heart failure has received extensive attention [26]. Emerging evidence confirmed that m^7^G methylation modification exerts critical functions in the development of immune-related diseases [27]. However, few studies have explored m^7^G methylation alterations in HF. Our study is the first to investigate the role of m^7^G regulators in HF and reveal the association between m^7^G methylation modifications and immune signatures. First, the expression of most m^7^G regulators were significantly different between HF patients and NFDs, and the expression levels correlated with immune cell infiltration and immune-related functions in HF. Second, using three machine learning algorithms, we established a five-gene m^7^G regulator diagnostic signature with excellent diagnostic performance for HF. Third, unsupervised cluster analysis of the HF samples using m^7^G regulators expression profiles led us to two m^7^G subtypes of HF with distinct m^7^G modification pattern and immune characteristics. Fourth, based on m^7^G-related DEGs, we further discovered two gene subgroups with unique m^7^G modification patterns and immune signatures, and WGCNA revealed m^7^G-related hub genes (UQCRC1, NDUFB6, and NDUFA13) with significant clinical relevance to cardiac function.

HF is a cardiovascular clinical syndrome with high morbidity and poor prognosis^28^. The pathogenesis of HF is complex and diverse, and so far, the molecular mechanism of HF has not been fully elucidated [29]. The development of cardiac remodeling of HF patients is accompanied by a higher inflammatory status, with fibrosis, cardiac cellular apoptosis, and modification of cardiac chambers morphology, volumetry and function, leading to depression of cardiac pump [30]. With the in-depth development of epigenetic research, more and more regulators of gene transcription and translation have been discovered, such as non-coding RNA (microRNAs, long non-coding RNAs, circular RNAs) and transcription factors (TFs). Among them, microRNAs can bind mRNAs at their 3´-UTRs, leading to mRNA degradation or inhibition of protein translation, and their regulatory effects on HF-related gene expression have been widely concerned and studied previously [31]. In addition, a series of RNA modifications including m^6^A, 5-methylcytosine (m^5^C), N^1^-adenosine methylation (m^1^A), N^4^-acetylcytidine (ac^4^C), 2'-O-methylation (2'O-Me), pseudouridine, and m^7^G in different RNA types have been widely implicated in various pathophysiological processes [4]. Regarding the development of HF, m^6^A methylation has been reported to be involved in cardiac function regulation by modulating calcium homeostasis [32], energy metabolism [33], translation process [34], autophagy [35], and so on. In addition, Nagasawa et al. reviewed that snoRNA-guided 2′O-Me can contribute to cardiac hypertrophy and HF through controlling mRNA transcript abundance and translation [36]. Therefore, RNA modifications may serve as new targets for the diagnosis and treatment of HF.

In recent years, m^7^G, a modification with a methyl group added to the 7th N-position of RNA guanine (G) in the 5′ cap region of tRNA, rRNA, and eukaryotic mRNA, has been receiving extensive attention as one of the most common forms of base modification in RNA post-transcriptional levels. Accumulating evidence suggests that m^7^G exerts an important role in regulating gene expression, mRNA splicing, transcription, and nuclear export of mRNA, as well as mRNA translation [37]. Zhao et al. reported that m^7^G methyltransferase METTL1 promotes post-ischemic angiogenesis via promoting VEGFA mRNA translation [11]. Dong et al. identified the m^7^G modification pattern (MGCS1, MGCS2, and MGCS3) in clear cell renal cell carcinoma by multi-omics analysis and characterized the correlation between this pattern and tumor microenvironment infiltration [27]. However, the role of m^7^G in the development of HF has been poorly reported. Therefore, in our study, we obtained 29 m^7^G RNA methylation regulators from the MSigDB team and systematically evaluated the modification pattern of m^7^G regulators in HF based on six microarray datasets. Among them, 14 regulators were differentially expressed between HF patients and NFDs, and correlation analysis showed strong correlations among the 14 m^7^G regulators. Taken together, the results highlight that m^7^G methylation modification patterns significantly differ between HF and NFD samples, and that m^7^G regulators may function as a complex to modulate the development of HF.

Based on the above conclusions, we further utilized machine learning algorithms to identify the m^7^G regulator diagnostic signature of HF. Machine learning has recently received extensive attention and applications in the field of bioinformatics due to its powerful data processing capabilities [38, 39]. Feature selection is a machine learning algorithm that determines the minimum set of relevant indicators required by a machine learning model. The biggest advantage of the algorithm is that it can remove redundant and irrelevant features, thereby reducing the input dimensionality, improving model accuracy, and reducing model complexity [40]. Of note, combined application of multiple feature selection algorithms has become an important method for disease-related molecular screening [41]. For example, in the study of Deng et al., SVM-RFE and LASSO were combined used to screen diagnostic markers for osteoarthritis [17]. Similarly, in our study, we combined three machine learning algorithms including BSR analysis, regularization techniques, and RF algorithm for feature selection. Regarding regularization techniques, three regression models (RIDGE, LASSO, and EN) were established based on the HF training dataset, and RIDGE regression model was identified as the optimal regularization model with the minimum value of RMSE in the internal validation dataset. Furthermore, intersection of the features from the three algorithms revealed that NUDT16, NUDT4, CYFIP1, LARP1, and DCP2 are potential m^7^G regulator diagnostic markers for HF. In addition, multivariate logistic regression analysis showed that the five-gene m^7^G regulator diagnostic signature exhibited a high degree of discrimination and calibration in predicting HF, which was further validated in two external validation datasets. Especially, through qRT-PCR experiments conducted in heart tissues and plasma samples, NUDT16, NUDT4, CYFIP1, and LARP1 were significantly downregulated in of HF patients compared with NFDs, which further strengthen the relevance of their biomarker discovery.

Emerging evidence suggests that immune and inflammatory activation plays a vital role in the progression of HF^2^. Under mechanical or chemical stimuli, cardiomyocytes can secrete inflammatory cytokines and chemokines to induce the activation of immune cells, fibroblasts, and pro-hypertrophic and pro-fibrotic signaling pathways, thereby inducing cardiac hypertrophy and triggering cardiac fibrosis and remodeling [42]. Accordingly, cardiac over-stretch related biomarkers, especially those associated with excessive cardiac inflammation, can be effective in predicting worse clinical outcomes and loss of therapeutic response in HF patients [43]. For example, Sardu et al. reported first time that RyR1 glycation in circulating lymphocytes represents a novel biomarker to predict CRT responsiveness [44]. Consistently, in the present study, we explored the immune characteristics in HF using the ssGSEA algorithm, and observed that the abundances of 12 immune infiltrating cells (aDCs, B cells, CD8 + T cells, iDCs, macrophages, mast cells, neutrophils, NK cells, Th1 cells, Th2 cells, TIL, and Treg) and 8 immune-related functions (APC co-inhibition, check-point, cytolytic activity, HLA, inflammation promoting, T cell co-inhibition, T cell co-stimulation, and type I IFN response) were significantly different between HF and NFDs samples. Furthermore, correlation analysis showed that the 14 differentially expressed m^7^G regulators were closely related to many immune characteristics. It is worth noting that CYFIP1-Treg was the most positively correlated m^7^G immune cell pair, and for immune-related functions, CYFIP1 was most positively correlated with parainflammation, suggesting that the CYFIP1-associated m^7^G regulatory pathway may be closely related to HF immunity. Besides, among the five diagnostic markers of m^7^G regulators, DCP2 was also found highly correlated with infiltration of various immune cells and immune-related functions, LARP1 was mainly associated with immune cell infiltration, and NUDT16 was mainly associated with immune-related functions. The above results suggest that these genes are likely to be involved in the progression of HF by regulating immune-related pathways. However, due to the lack of clinical characteristics of HF patients in the included studies, such as changes in BNP values, the specific relationship between m^7^G regulators changes and excessive activation of inflammation and the degree of HF, needs to be explored in subsequent clinical studies.

In our study, unsupervised clustering of the HF samples using differentially expressed m^7^G regulators expression profiles led us to two HF subtypes with distinctive m^7^G modification pattern. As for immune characteristics, m^7^G subtype A of HF was observed with a higher abundance of immune-related functions including CCR and T cell co-stimulation, while m^7^G subtype B was characterized by increased infiltration levels of B cells. B cells have been previously reported to play a crucial role in the progression of HF through direct regulation of antibody secretion and indirect regulation of antigen presentation and cytokine/chemokine secretion [45]. Our classification strategy can help us understand the underlying mechanisms of immune regulation in HF, and thereby apply m^7^G-related intervention strategies to the precision treatment of HF. Additionally, enrichment analysis showed that the m^7^G subtype-related DEGs were mainly enriched in cardiac function-associated biological processes, as well as heart-related and metabolism-related pathways, indicating that m^7^G methylation may server as a key target in regulating cardiomyocyte material and energy metabolism.

Based on the m^7^G subtype-associated DEGs, two distinct gene subgroups of HF patients were identified with similar grouping trend to the m^7^G subtype clustering. In addition, using WGCNA and the cytoHubba plugin, we screened ten m^7^G methylation-related hub genes in HF, among which UQCRC1, NDUFB6, and NDUFA13 were positively correlated with LVEF of HF patients. UQCRC1 is a key subunit of complex III of the mitochondrial respiratory chain, which plays a critical role in electron transport and ATP generation [46]. High expression of UQCRC1 leads to mitochondrial dysfunction and reduced ATP utilization, thereby accelerating the process of lipid deposition and insulin resistance in skeletal muscle, ultimately leading to the development of type 2 diabetes and obesity [47]. NDUFA13 encodes a subunit of mitochondrial complex I, and its downregulation creates a leak within complex I, which significantly suppresses the superoxide burst and eventually dampens myocardial ischemia and reperfusion injury [48]. Likewise, NDUFB6 is an accessory subunit of mitochondrial complex I and has been reported to be required for electron transfer activity in cell energy metabolism [49]. These results suggest that these m^7^G methylation modification markers may be involved in the development of HF through regulating the mitochondrial respiratory chain.

Our study has limitations that must be acknowledged. First, the sample size of HF patients in the GEO database is relatively small. Although we have included as many studies that met the criteria as possible and eliminated batch effects to integrate information from different datasets, studies with larger sample sizes are still needed in subsequent analysis and validation. Second, clinical information of HF patients such as heart failure grading, treatment, and prognosis cannot be obtained. Subsequently, the correlation between m^7^G patterns and clinical characteristics or prognosis cannot be analyzed. Third, this study is mostly based on bioinformatics analysis, although experimental validation of m^7^G regulator expression has been performed in heart tissues and plasma of HF and NFD subjects. Fourth, there is a lack of downstream evidence of the m^7^G modifications among HF and NFD subjects, and the in-depth mechanism and pathway of m^7^G methylation regulating myocardial energy metabolism and immune infiltration need to be further investigated in follow-up studies. Fifth, modifications of cardiac remodeling in HF could pass toward different expression of a cluster of epigenetic regulators such as the miRNAs and TFs [31]. Due to the lack of relevant data in the included studies, the correlation analysis between m^7^G regulators and epigenetic factors was not carried out in our study, which should be improved and explored in subsequent molecular studies of advanced HF.

## Conclusions

In conclusion, to the best of our knowledge, this is the first study to explore the role of m^7^G RNA methylation in HF. Through cross-combination of three machine learning methods, we established a five-gene m^7^G regulator diagnostic signature with excellent discrimination and calibration to distinguish HF and NFD samples. Based on the differentially expressed m^7^G regulators, two distinct m^7^G subtypes and gene subgroups in HF patients were identified with significant differences in m^7^G regulators expression, immune characteristics, and biological functions. Additionally, we revealed an association between m^7^G subtypes and immune signatures, which can be used to guide future immunotherapy of HF. Moreover, three m^7^G methylation-related hub genes were identified as significantly correlated with LVEF in HF patients, suggesting that they may serve as indicators of disease severity in HF.

## Methods

### Dataset and preprocessing

The research strategy is presented in Fig. 1. The GEO database (www.ncbi.nlm.nih.gov/geo/) was searched to obtain the datasets based on the search terms of “heart failure” or/and “HF.” Eligible datasets were selected according to the following criteria: (i) the organism was filtered as “homo sapiens”; (ii) the study type was set as “Expression profiling by array”; (iii) array data for both HF and NFDs should be included in the dataset and at least three heart samples were investigated; (iv) the raw data should be provided for reanalysis. Six datasets were finally included: GSE16499 [18], GSE26887 [19], GSE42955 [20], GSE57338 [21], GSE76701 [22], and GSE79962 [23]. In total, 124 HF patients and 135 NFDs were included in our study to evaluate the expression levels of m^7^G regulators. Raw CEL files of these datasets and their corresponding platform files were downloaded to construct the gene expression profile. Through the limma Bioconductor package [50], the six raw datasets were preprocessed via background adjustment and quantile normalization. Then, the “Combat” algorithm in the sva R package was used to correct for batch effects between these six datasets due to different platforms, different labs and different time points, and the results with and without the adjustment were visualized as PCA plots, respectively (Additional file 1: Fig. S6). After applying batch correction, six datasets were combined into a merged dataset for further analysis. In addition, GSE46224 [24] (15 HF patients, 8 NFDs) and GSE116250 [25] (50 HF patients, 14 NFDs) were used as external validation RNA-seq datasets. The characteristics of these eight datasets are summarized in Additional file 1: Table S2 and Additional file 1: Table S3.

### Identification of differentially expressed m^7^G regulator genes

The m^7^G regulator genes were retrieved from the Molecular Signatures Database (MSigDB, http://software.broadinstitute.org/gsea/msigdb, Additional file 1: Table S4). Based on these m^7^G regulator genes, the R package “limma” was applied to determine DEGs between HF and NFDs samples. Then, the regulatory relationship among the differentially expressed m^7^G regulator genes was evaluated by correlation analysis.

### Development and validation of m^7^G regulator diagnostic signature for HF

In order to develop a m^7^G-related diagnostic signature, feature selection was conducted based on the differentially expressed m^7^G regulator genes. First, the merged dataset was divided into a training dataset (70%) and an internal validation dataset (30%), and tenfold cross-validation was employed to prevent overfitting of the model. Second, BSR analysis was used from the R package “leaps” to identify biomarkers that best predicted HF. Third, using the “glmnet” package, three regularized linear methods, including RIDGE regression, LASSO regression and EN regression were applied to identify which variables contributed most to the estimate prediction of HF. All the models were developed on the training dataset. Model performance was further evaluated by RMSE in the internal validation dataset, and genes from the best performing model were obtained. Fourth, based on the RF algorithm, the R package “randomForest” was used for feature selection and construction of the diagnostic signature. Eventually, we took the intersection of genes from BSR analysis, regularized linear regression, and RF algorithm for further analysis.

Diagnostic model of HF was further built by fitting the intersected m^7^G regulator genes into a binary logistic regression model (glm package, *R*). The *R* packages “rms” and “forestplot” were used to construct the nomogram and forest plot, respectively. The discriminatory capability of the model was assessed using the receiver operating characteristic (ROC) curve, and the calibration was evaluated with a calibration plot. The evaluation was conducted in the merged dataset and two external validation datasets.

### Validation of m^7^G regulator expression in human samples using qRT-PCR experiments

For further validation, heart tissues and plasma samples from HF patients and NFDs were obtained for qRT-PCR validation experiments. Five mL of whole blood sample was collected with anticoagulant (EDTA)-treated tube. Blood sample was centrifuged in primary blood collection tubes for 10 min at 1500 × g and 4 °C using a swinging bucket rotor. The upper (yellow) plasma phase was carefully transferred to a new tube without disturbing the intermediate buffy coat layer (containing white blood cells and platelets). The plasma sample was further centrifuged in conical tubes for 10 min at 12,000 × g and 4 °C to remove additional cellular nucleic acids attached to cell debris. Following centrifugation, the cleared supernatant (plasma) should be immediately transferred to a new tube without disturbing the pellet. The plasma samples should be maintained at 2–8 °C while handling or stored at − 80 °C. Heart tissues from HF patients and NFDs were obtained from the Specimen Bank of Cardiovascular Surgery Laboratory and Department of Pathology of Shanghai Changhai Hospital, China. Written informed consents were obtained from all patients or family members, and the study was approved by the institute ethics committee of Changhai Hospital.

Total RNAs from heart tissues or plasma samples were isolated using Trizol reagent (Trizol™ Reagent, Invitrogen) or miRNeasy Serum/Plasma Kit (Qiagen, Cat. No. 217184), separately. RNAs were then reverse-transcribed into cDNAs using TOYOBO ReverTra Ace®qRT-PCR RT Kit (TOYOBO, Japan). SYBR®GREEN (TOYOBO, Japan) was used for qRT-PCR, and the primer sequences used are listed as follows: CYFIP1 forward, 5’-CAGGTGGTTCCGCTATTTGG-3’ and reverse, 5’-ATGTTGTACTGAGGGCTGCT-3’; LARP1 forward, 5’-ACGAGGAGATGGAGCAGATG-3’ and reverse, 5’-GCGCATGTAATGTGGTGTCT-3’; DCP2 forward, 5’-AGACCAAACGGGTGGAGATT-3’ and reverse, 5’-TCCTCGCTGGGAATATGCAA-3’; NUDT4 forward, 5’-GCTAAAGCTGGGTTGTTCCC-3’ and reverse, 5’-TAGATGGCAACCCAGAGGTC-3’; NUDT16 forward, 5’-CGGGAGCAGTTACTTGAAGC-3’ and reverse, 5’-GGCCTGAAATAGAGCCAGAC-3’. The expression levels of mRNAs relative to glyceraldehyde-3-phosphate dehydrogenase (GAPDH) or external reference were detected using the 2^–ΔΔCt^ method.

### Correlation analysis between m^7^G regulators and immune characteristics

To evaluate the characteristics of the immune infiltration in HF, the ssGSEA method was used to explore the different infiltration degrees of 29 immune characteristics (16 immune cell types and 13 immune-related functions) in HF patients and NFDs samples. In addition, correlation analysis was used to determine the association between m^7^G regulators and immune characteristics in HF, which was visualized by correlation heatmaps.

### Consensus clustering analysis of the differentially expressed m^7^G regulator genes

Based on the differentially expressed m^7^G regulator genes, consensus clustering analysis was performed to categorize HF patients into distinct molecular subtypes using the *R* package “ConsensusClusterPlus,” and the model was run through a total of 1000 iterations to ensure the stability of these categories. In addition, GSVA was conducted with the KEGG gene set of “c2.cp.kegg.v7.4.-symbols” to determine the significant pathways among these m^7^G regulator genes subtypes. Finally, immune-related signature of the different subtypes was further constructed.

### Recognition of DEGs associated with the m^7^G subtypes

Using an empirical Bayesian method through the R package “limma,” DEGs among the m^7^G subtypes were calculated with an absolute log fold change (l*ogFC*) > 1 and an adjusted *p*-value < 0.05. Gene enrichment analysis of these DEGs was performed using the Clusterprofiler Bioconductor package, including biological processes (BP), cellular component (CC), and molecular function (MF) terms in GO functional and KEGG pathways enrichment.

### Construction of HF gene subgroups based on DEGs

To further explore the potential biological behavior of each m^7^G pattern, consensus clustering was performed to divide HF patients into distinct gene subgroups based on the expression of m^7^G subtype-related DEGs. The algorithm was repeated for 1000 cycles to guarantee the robustness of the clustering. Additionally, we analyzed the difference of infiltration of immune cells and immune-related functions in different gene subgroups.

### Identification of m^7^G methylation-related hub genes in HF

To identify genes correlating with disease activity of HF, we performed WGCNA based on the gene profiles and gene subgroups of HF patients. First, the genes with upper 25% median absolute deviation across all samples in the integrated dataset were selected to guarantee data heterogeneity and accuracy of WGCNA. Second, samples located in the clusters and passed the cutoff thresholds were included in the subsequent analysis. Third, the adjacency matrix was calculated and then converted into a topological overlap matrix (TOM) with the soft thresholding power β. Fourth, according to the TOM-based dissimilarity measure, genes were divided into different gene modules using the dynamic tree cut algorithm, and modules whose eigengenes were highly correlated (correlation greater than 0.8) were merged. Finally, the co-expressed genes were determined by calculating the module membership (MM) and gene significance (GS) of the genes in the target modules.

In addition, we used the search tool for the retrieval of interacting genes/proteins (STRING) online database (https://string-db.org/) to construct protein–protein interaction (PPI) networks of the co-expressed genes. A threshold weight of 0.7 was used for selecting the connection between two proteins. We extracted the largest connected component of the PPI network and computed the betweenness using Cytoscape. Hub genes, top 10 genes with the highest degree, were then identified using the plugin CytoHubba. Furthermore, correlations between the expression profiles of these hub genes and LVEF of HF patients were determined using the GSE46224 dataset.

### Statistical analyses

Comparisons between two groups were evaluated with Student’s t test (for normally distributed data with equal variance) or Wilcoxon’s test (for non-normally distributed data). All statistical tests were two-sided, and the significance level was set at *p* < 0.05. Pearson correlation coefficient was calculated when variables were normally distributed, otherwise Spearman's rank correlation coefficient was calculated. Correlation coefficients < 0.3 were considered negligible, and statistical significance was indicated by *p* < 0.05 [51]. All statistical data analyses were implemented using *R* (version 4.1.2) and *R* studio (version 2021.9.1).

## Supplementary Information

**Additional file 1: Table S1.** Association of HF occurrence with the differential expression of the five m^7^G regulator markers. **Table S2.** The characteristics of six screened microarray datasets of HF in GEO database. **Table S3.** The characteristics of two validation RNA-seq datasets of HF in GEO database. **Table S4.** Summary of the 29 m^7^G RNA methylation regulator genes. **Fig. S1.** External validation of the diagnostic value of m^7^G regulator signature for HF. **Fig. S2.** Unsupervised consensus clustering analysis for HF samples based on m^7^G regulators expression profiles. **Fig. S3.** The expression profiles of the 14 m^7^G regulators in HF subtype A, subtype B, and NFDs were compared using one way ANOVA. **Fig. S4.** The distribution of infiltrating immune cells or activity of immune-related functions in each HF subtype was compared with that of NFDs. **Fig. S5.** Clustering analysis for HF samples based on the m^7^G subtype-related differentially expressed genes. **Fig. S6.** Principal component analysis of batch‐corrected expression data of HF microarray datasets.

## Data Availability

The datasets presented in this study can be found in online repositories. The names of the repository/repositories and accession number(s) can be found in the article/supplementary material. Specific data produced in this study will be available from the authors on request without restriction.

## Abbreviations

HF: Heart failure
m^6^A: N^6^-methyladenosine
m^7^G: N^7^-methylguanosine
tRNA: Transfer RNA
rRNA: Ribosomal RNA
GEO: Gene expression omnibus
LASSO: Least absolute shrinkage and selection operator
SVM-RFE: Support vector machine-recursive feature elimination
NFDs: Nonfailing donors
DEGs: Differentially expressed genes
BSR: Best subset regression
EN: Elastic net
RMSE: Root mean squared error
RF: Random forest
qRT-PCR: Reverse-transcription polymerase chain reaction
ROC: Receiver operating characteristic
ssGSEA: Single-sample gene-set enrichment analysis
GSVA: Gene-set variation analysis
KEGG: Kyoto Encyclopedia of Genes and Genomes
GO: Gene ontology
BP: Biological processes
CC: Cellular component
MF: Molecular function
WGCNA: Weighted gene co-expression network analysis
TOM: Topological overlap matrix
MM: Module membership
GS: Gene significance
STRING: Search tool for the retrieval of interacting genes/proteins
PPI: Protein–protein interaction
LVEF: Left ventricular ejection fraction
BIC: Bayesian information criterion
