# Supplementary material for: Identification of m7G regulator-mediated RNA methylation modification patterns and related immune microenvironment regulation characteristics in heart failure
Source: Clin Epigenetics. 2023 Feb 13;15:22. doi: 10.1186/s13148-023-01439-3 (PMC9926673; doi:10.1186/s13148-023-01439-3)
Supplement: Supplementary file 1 — Additional file 1: Table S1. Association of HF occurrence with the differential expression of the five m7G regulator markers. Table S2. The characteristics of six screened microarray datasets of HF in GEO database. Table S3. The characteristics of two validation RNA-seq datasets of HF in GEO database. Table S4. Summary of the 29 m7G RNA methylation regulator genes. Fig. S1. External validation of the diagnostic value of m7G regulator signature for HF. Fig. S2. Unsupervised consensus clustering analysis for HF samples based on m7G regulators expression profiles. Fig. S3. The expression profiles of the 14 m7G regulators in HF subtype A, subtype B, and NFDs were compared using one way ANOVA. Fig. S4. The distribution of infiltrating immune cells or activity of immune-related functions in each HF subtype was compared with that of NFDs. Fig. S5. Clustering analysis for HF samples based on the m7G subtype-related differentially expressed genes. Fig. S6. Principal component analysis of batch‐corrected expression data of HF microarray datasets. [file 13148_2023_1439_MOESM1_ESM.pdf]

Supplementary Information for

Identification of m^{7}G regulator-mediated RNA methylation modification patterns and related immune microenvironment regulation characteristics in heart failure

Table S1. Association of HF occurrence with the differential expression of the five m^{7}G regulator markers
Table S2. The characteristics of six screened microarray datasets of HF in GEO database
Table S3. The characteristics of two validation RNA-seq datasets of HF in GEO database
Table S4. Summary of the 29 m^{7}G RNA methylation regulator genes

Fig. S1. External validation of the diagnostic value of m^{7}G regulator signature for HF
Fig. S2. Unsupervised consensus clustering analysis for HF samples based on m^{7}G regulators expression profiles
Fig. S3. The expression profiles of the 14 m^{7}G regulators in HF subtype A, subtype B, and NFDs were compared using one way ANOVA.
Fig. S4. The distribution of infiltrating immune cells or activity of immune-related functions in each HF subtype was compared with that of NFDs.
Fig. S5. Clustering analysis for HF samples based on the m^{7}G subtype-related differentially expressed genes
Fig. S6. Principal component analysis of batch-corrected expression data of HF microarray datasets

References
Abbreviations: RNA-seq, RNA-sequencing; HF, heart failure; NFD, nonfailing donor; GEO, Gene Expression Omnibus; OR, odds ratio.

Table S1 Association of HF occurrence with the differential expression of the five m^{7}G regulator markers

ID	OR	OR 0.95L	OR 0.95H	*p*-value
NUDT16	-3.2375	-4.0845	-2.3905	0.000132
NUDT4	-2.9628	-3.4544	-2.4712	1.67E-09
CYFIP1	-4.9084	-5.8093	-4.0075	5.08E-08
LARP1	-3.5071	-4.6744	-2.3398	0.00266
DCP2	4.4154	3.3387	5.4921	4.12E-05

Table S2. The characteristics of six screened microarray datasets of HF in GEO database

Dataset ID	Country	Number of samples		Microarray platform
		HF	NFD	
GSE16499(1)	USA	15	15	Affymetrix Human Exon 1.0 ST Array
GSE26887(2)	Italy	7	5	Affymetrix Human Gene 1.0 ST Array
GSE42955(3)	Spain	24	5	Affymetrix Human Gene 1.0 ST Array
GSE57338(4)	USA	54	95	Affymetrix Human Gene 1.1 ST Array
GSE76701(5)	USA	4	4	Affymetrix Human Genome U133 Plus 2.0 Array
GSE79962 (6)	USA	20	11	Affymetrix Human Gene 1.0 ST Array

Table S3. The characteristics of two validation microarray datasets of HF in GEO database

Dataset ID	Country	Number of samples		Platform ID
		HF	NFD	
GSE46224(7)	USA	15	8	GPL11154
GSE116250(8)	USA	50	14	GPL16791

Table S4 Summary of 29 m^7G RNA methylation regulator genes

Gene	Type
METTL1	m^7G RNA methylation regulators
WDR4	m^7G RNA methylation regulators
NSUN2	m^7G RNA methylation regulators
DCP2	m^7G RNA methylation regulators
DCPS	m^7G RNA methylation regulators
NUDT10	m^7G RNA methylation regulators
NUDT11	m^7G RNA methylation regulators
NUDT16	m^7G RNA methylation regulators
NUDT3	m^7G RNA methylation regulators
NUDT4	m^7G RNA methylation regulators
NUDT4B	m^7G RNA methylation regulators
AGO2	m^7G RNA methylation regulators
CYFIP1	m^7G RNA methylation regulators
EIF4E	m^7G RNA methylation regulators
EIF4E1B	m^7G RNA methylation regulators
EIF4E2	m^7G RNA methylation regulators
EIF4E3	m^7G RNA methylation regulators
GEMIN5	m^7G RNA methylation regulators
LARP1	m^7G RNA methylation regulators
NCBP1	m^7G RNA methylation regulators
NCBP2	m^7G RNA methylation regulators
NCBP3	m^7G RNA methylation regulators
EIF3D	m^7G RNA methylation regulators
EIF4A1	m^7G RNA methylation regulators
EIF4G3	m^7G RNA methylation regulators
IFIT5	m^7G RNA methylation regulators
LSM1	m^7G RNA methylation regulators
NCBP2L	m^7G RNA methylation regulators
SNUPN	m^7G RNA methylation regulators

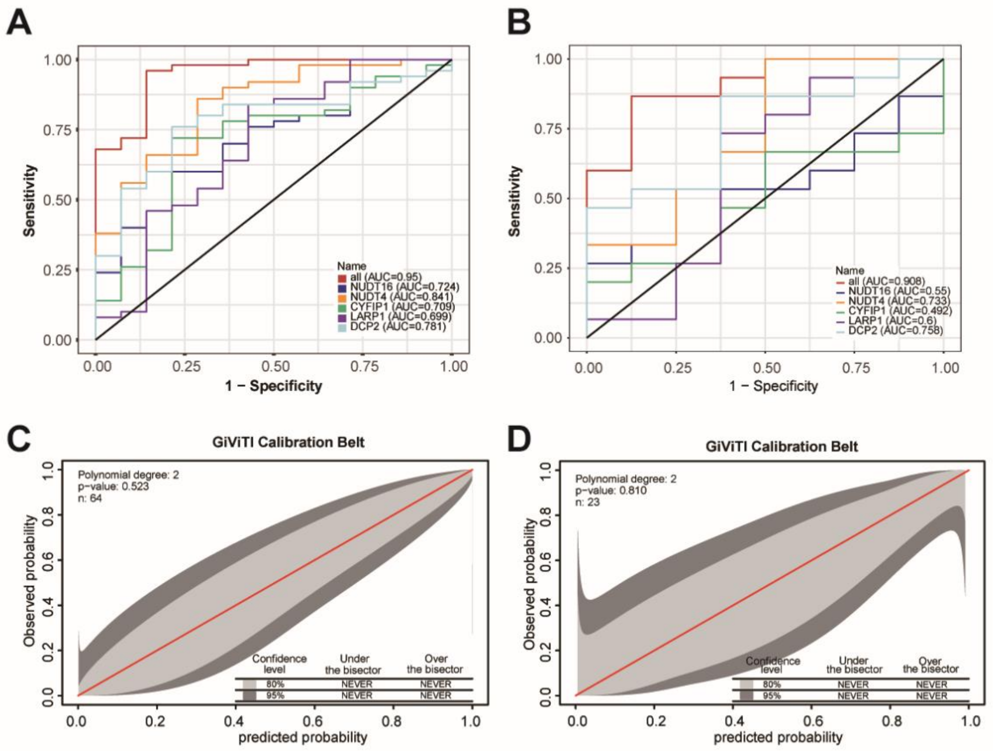

Fig. S1 External validation of the diagnostic value of m7G regulator diagnostic signature for HF. A-B, Five m^{7}G regulators, including NUDT16, NUDT4, CYFIP1, ARP1, and DCP2, were analyzed for their ability to discriminate between NFD and HF samples by ROC curves in two validation RNA-seq datasets, including GSE46224 (A) and GSE116250 (B). C-D, Calibration curve of the five-gene m^{7}G regulator diagnostic signature in two validation RNA-seq datasets, including GSE46224 (C) and GSE116250 (D).

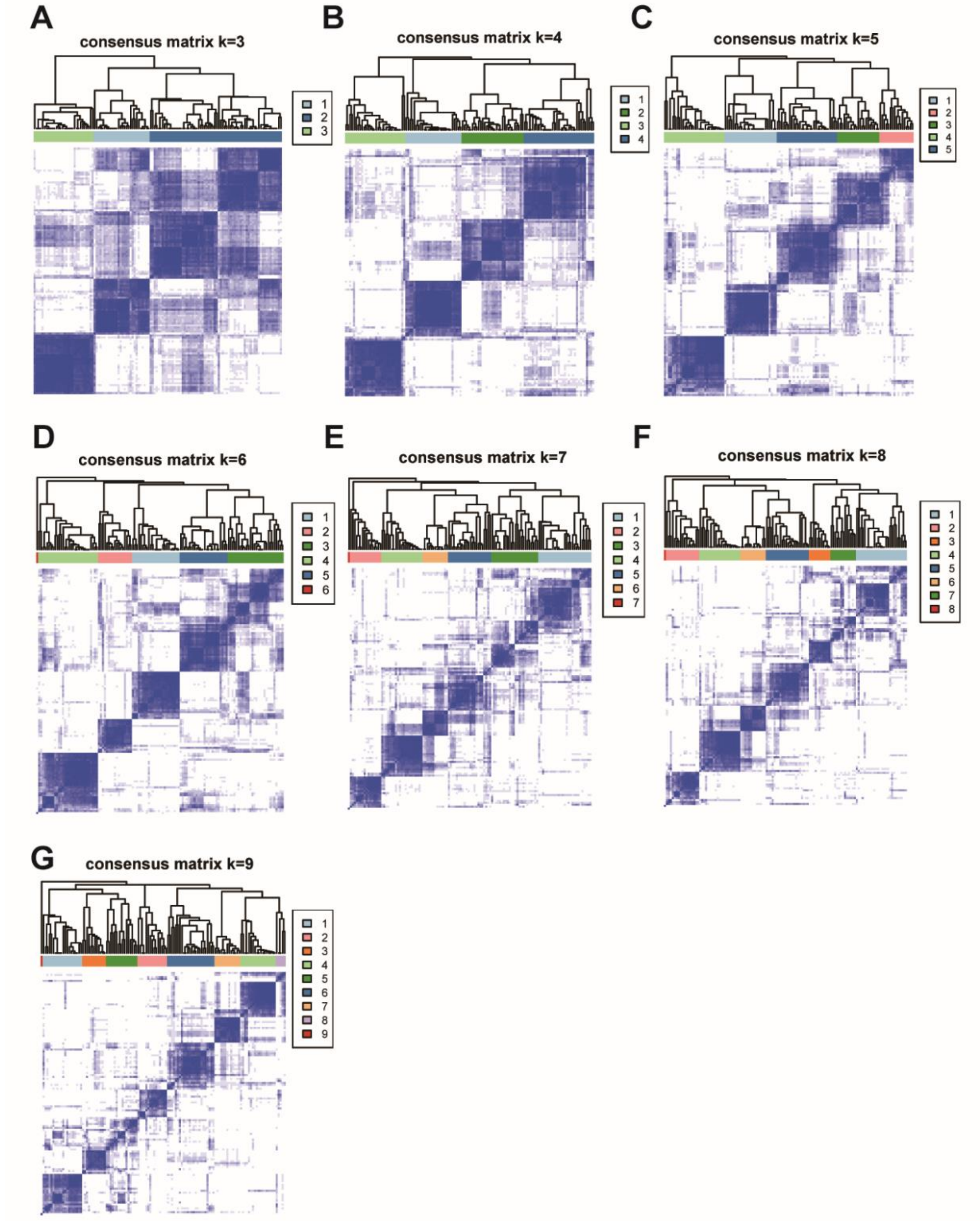

Fig. S2 Unsupervised consensus clustering analysis for HF samples based on m^{7}G regulators expression profiles. A-G, Heatmaps of the matrix of co-occurrence proportions for k = 3-9.

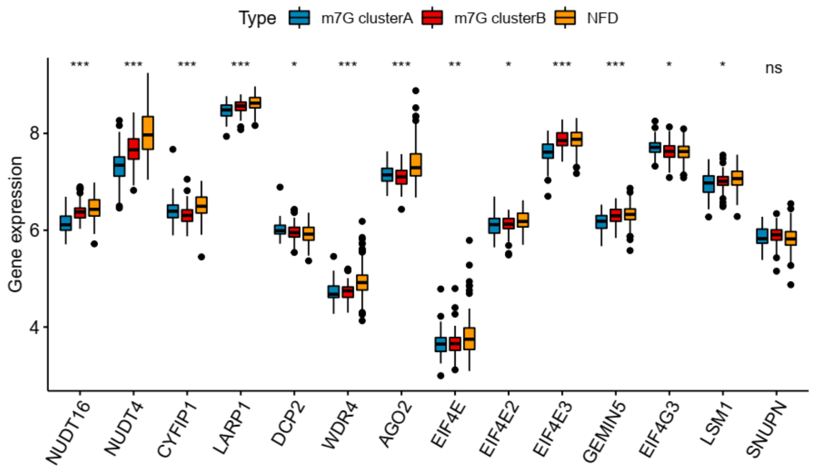

Fig. S3. The expression profiles of the 14 m^{7}G regulators in HF subtype A, subtype B, and NFDs were compared using one way ANOVA. ns = not significant, *$p < 0.05$, **$p < 0.01$, and ***$p < 0.001$ among the three groups.

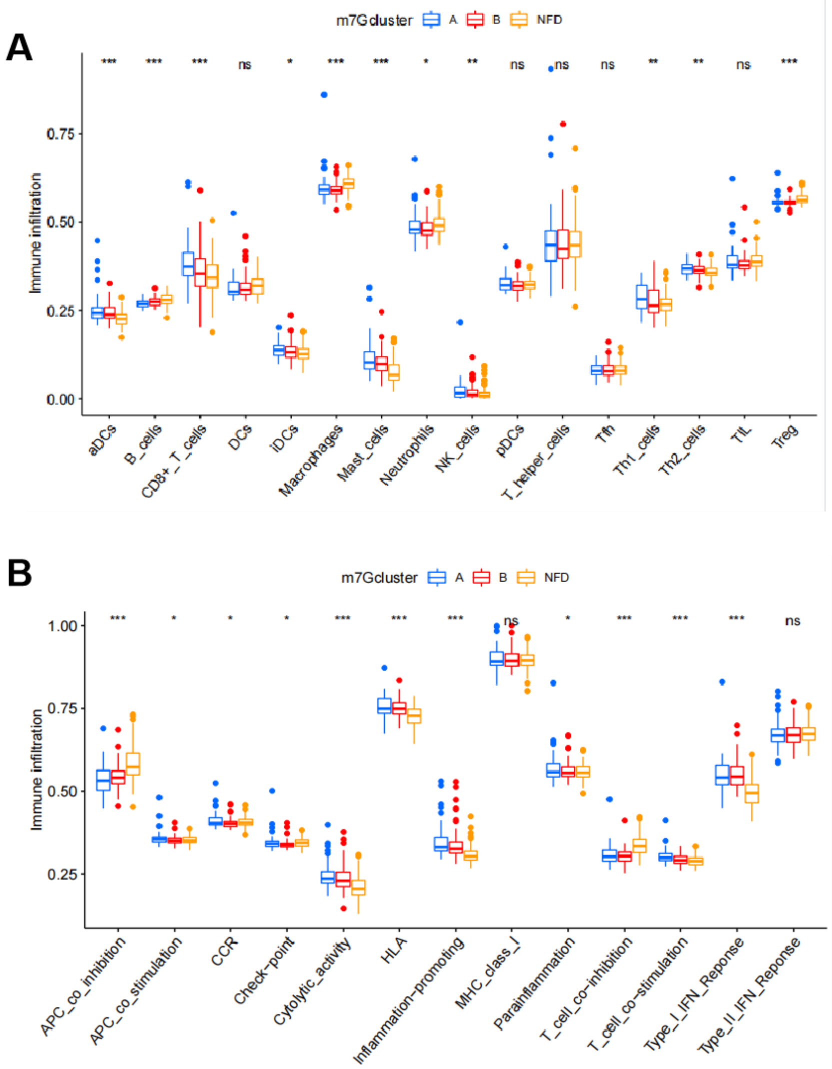

Fig. S4. The distribution of infiltrating immune cells or activity of immune-related functions in each HF subtype was compared with that of NFDs. A, The infiltration scores of 16 immune cells among two m^{7}G subtypes and NFDs. One way ANOVA was used. ns = not significant, *$p < 0.05$, **$p < 0.01$, and ***$p < 0.001$ among the three groups. B, The infiltration scores of 13 immune-related functions among two m^{7}G subtypes and NFDs. ns = not significant, *$p < 0.05$, ***$p < 0.001$ among the three groups.

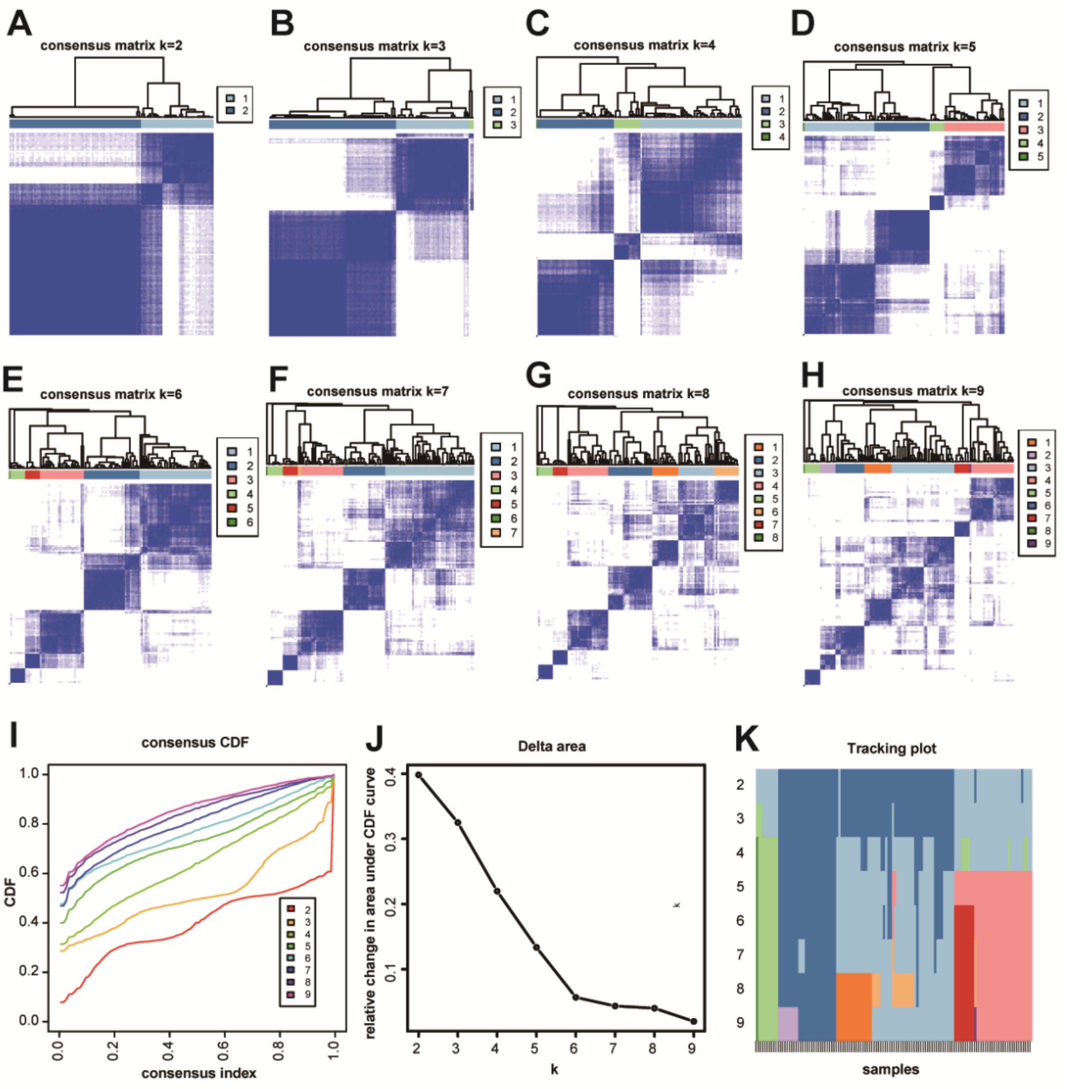

Fig. S5 Clustering analysis for HF samples based on the m^7G subtype-related differentially expressed genes. A-H, Heatmap of the matrix of co-occurrence proportions for HF samples for k = 2-9. I, Consensus clustering cumulative distribution function (CDF) for k = 2-9. J, Relative change in area under CDF curve for k = 2-9. K, Tracking plot for k = 2-9.

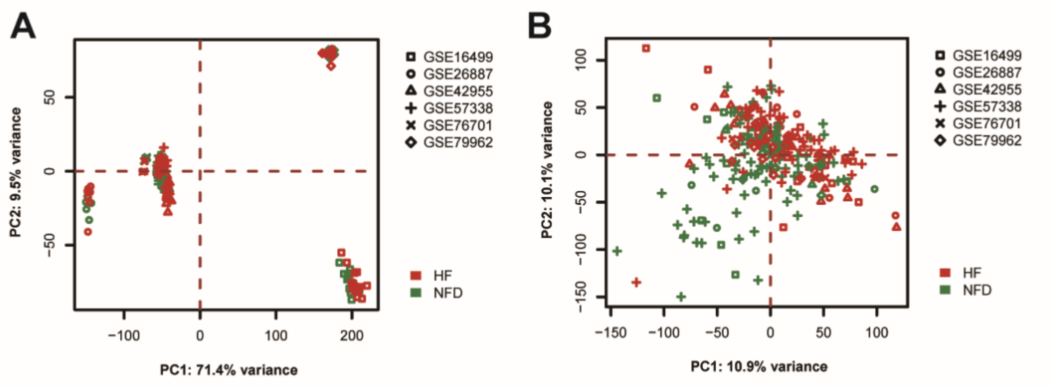

Fig. S6 Principal component analysis of batch-corrected expression data of HF microarray datasets. The gene expression profiling was annotated using the annotation document of corresponding platforms, and the batch effects were eliminated by implementation of the “Combat” algorithm in the sva R package. A, Before batch correction; B, After batch correction.

References

1. Kong SW, Hu YW, Ho JW, Ikeda S, Polster S, John R, et al. Heart failure-associated changes in RNA splicing of sarcomere genes. Circulation Cardiovascular genetics. 2010;3(2):138-46.
2. Greco S, Fasanaro P, Castelvecchio S, D'Alessandra Y, Arcelli D, Di Donato M, et al. MicroRNA dysregulation in diabetic ischemic heart failure patients. Diabetes. 2012;61(6):1633-41.
3. Molina-Navarro MM, Roselló-Lletí E, Ortega A, Tarazón E, Otero M, Martínez-Dolz L, et al. Differential gene expression of cardiac ion channels in human dilated cardiomyopathy. PloS one. 2013;8(12):e79792.
4. Liu Y, Morley M, Brandimarto J, Hannenhalli S, Hu Y, Ashley EA, et al. RNA-Seq identifies novel myocardial gene expression signatures of heart failure. Genomics. 2015;105(2):83-9.
5. Kim EH, Galchev VI, Kim JY, Misek SA, Stevenson TK, Campbell MD, et al. Differential protein expression and basal lamina remodeling in human heart failure. Proteomics Clinical applications. 2016;10(5):585-96.
6. Matkovich SJ, Al Khiami B, Efimov IR, Evans S, Vader J, Jain A, et al. Widespread Down-Regulation of Cardiac Mitochondrial and Sarcomeric Genes in Patients With Sepsis. Critical care medicine. 2017;45(3):407-14.
7. Yang KC, Yamada KA, Patel AY, Topkara VK, George I, Cheema FH, et al. Deep RNA sequencing reveals dynamic regulation of myocardial noncoding RNAs in failing human heart and remodeling with mechanical circulatory support. Circulation. 2014;129(9):1009-21.
8. Sweet ME, Cocciolo A, Slavov D, Jones KL, Sweet JR, Graw SL, et al. Transcriptome analysis of human heart failure reveals dysregulated cell adhesion in dilated cardiomyopathy and activated immune pathways in ischemic heart failure. BMC Genomics. 2018;19(1):812.
